# Co‐delivery Nano System of MS‐275 and V‐9302 Induces Pyroptosis and Enhances Anti‐Tumor Immunity Against Uveal Melanoma

**DOI:** 10.1002/advs.202404375

**Published:** 2024-06-18

**Authors:** Hong Ren, Zhenkai Wu, Jia Tan, Hui Tao, Wangyuan Zou, Zheng Cao, Binyu Wen, Ziyi Cai, Jiaqi Du, Zhihong Deng

**Affiliations:** ^1^ Department of Ophthalmology The Third Xiangya Hospital Central South University Changsha Hunan 410013 China; ^2^ Department of Ophthalmology Changde Hospital Xiangya School of Medicine Central South University Changde Hunan 415000 China; ^3^ Department of Ophthalmology The first people's hospital of Changde city Changde Hunan 415000 China; ^4^ Eye Center of Xiangya Hospital Central South University Changsha Hunan 410008 China; ^5^ Hunan Key Laboratory of Ophthalmology and National Clinical Research Center for Geriatric Disorders Xiangya Hospital Central South University Changsha Hunan 410008 China; ^6^ Department of Anesthesiology Xiangya Hospital Central South University Changsha Hunan 410008 China; ^7^ Department of Chemical and Biomolecular Engineering University of California Los Angeles CA 90066 USA

**Keywords:** epigenetic therapy, immunotherapy, metabolic intervention, pyroptosis, uveal melanoma

## Abstract

In the treatment of uveal melanoma (UVM), histone deacetylase inhibitors (HDACi) have emerged as a promising epigenetic therapy. However, their clinical efficacy is hindered by the suboptimal pharmacokinetics and the strong self‐rescue of tumor cells. To overcome these limitations, reactive oxygen species (ROS)‐responsive nanoparticles (NPs) are designed that encapsulate HDACi MS‐275 and the glutamine metabolism inhibitor V‐9302. Upon reaching the tumor microenvironment, these NPs can disintegrate, thereby releasing MS‐275 to increase the level of ROS and V‐9302 to reduce the production of glutathione (GSH) related to self‐rescue. These synergistic effects lead to a lethal ROS storm and induce cell pyroptosis. When combined with programmed cell death protein 1 monoclonal antibodies (*α*‐PD‐1), these NPs facilitate immune cell infiltration, improving anti‐tumor immunity, converting “immune‐cold” tumors into “immune‐hot” tumors, and enhancing immune memory in mice. The findings present a nano‐delivery strategy for the co‐delivery of epigenetic therapeutics and metabolic inhibitors, which induces pyroptosis in tumors cells and improves the effectiveness of chemotherapy and immunotherapy.

## Graphical abstract

The co‐delivery of histone deacetylase inhibitor MS‐275 and glutamine metabolism inhibitor V‐9302 via nanomaterials surpasses traditional monotherapy limitations in treating uveal melanoma, inducing ROS storms and achieving unexpected pyroptosis. This study presents an effective nanomedicine that elicits a potent immune response through the induction of pyroptosis, resulting in ideal coordination capacity.

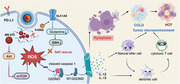

## 1 Introduction

Uveal melanoma (UVM), the most common intraocular malignancy in adults, presents a formidable clinical challenge due to its high mortality rate, 45% within 15‐years and its propensity for metastasis.^[^ ^1^ ^]^ Standard treatments such as surgery and radiotherapy, while effective for local tumors during early stages, fails to treat micro‐metastases and may cause damage to the surrounding eye tissues.^[^ ^2^ ^]^ Cancer immunotherapy has emerged as a transformative approach, engaging the body's host immune system to recognize and eliminate tumor cells precisely.^[^ ^3^ ^]^ However, the unique characteristics of UVM, including low mutational load, poor immunogenicity, and the immunosuppressive tumor microenvironment, compromise the effectiveness of immunotherapy.^[^ ^4^, ^5^ ^]^ Developing strategies that increase tumor immunogenicity and enhance the responses of UVM to immunotherapy is of great significance.

Epigenetic therapies have emerged as potential options to enhance the immunogenicity of cancer cells and improve anti‐tumor immunity, with histone deacetylase inhibitors (HDACi) at the forefront.^[^ ^6^, ^7^, ^8^ ^]^ Despite their promise, the performance of HDACi in treating solid tumors is often unsatisfactory.^[^ ^9^, ^10^, ^11^ ^]^ Monotherapy of HDACi in UVM is limited due to dose‐dependency, cellular metabolism, and cellular acetylation flux of the cell.^[^ ^12^, ^13^ ^]^ Preclinical studies suggested that, the high reactive oxygen species (ROS) environment in tumor cells usually results in activation of the mTOR pathway, which promotes glutamine uptake to generate glutathione (GSH) in order to sufficiently keep the cellular redox state.^[^ ^14^ ^]^ Notably, HDACi can disrupt the mTOR pathway but hard to cope with the uptake of glutamine by tumor cells.^[^ ^12^, ^15^, ^16^, ^17^, ^18^ ^]^ Such GSH‐associated ROS detoxification substantially limits the efficacy HDACi. Considering glutamine is essential for the biosynthesis of GSH in cancer cells, we hypothesize that employing HDACi to interfere with the mTOR pathway and concurrently inhibiting glutamine uptake can induce an uncontrollable ROS storm beyond the limit of a single‐agent action.

Pyroptosis, a pro‐inflammatory form of cell death, could serve as a linchpin in this strategy. Canonical pyroptosis is characterized by the cleavage of Gasdermin D (GSDMD) to form the N‐terminal of GSDMD (NT‐GSDMD) through cysteine asparaginase‐1, which perforates the cell membrane and releases pro‐inflammatory cytokines.^[^ ^19^ ^]^ It has been reported that ROS storm can contribute to both NT‐GSDMD oligomerization and pore‐forming activity, leading to enhanced pyroptosis.^[^ ^20^, ^21^, ^22^, ^23^ ^]^ Therefore, combining HDACi with metabolic inhibitors to simultaneously inhibit the mTOR activity and glutamine uptake can induce the ROS storm, resulting in cancer cell pyroptosis and promoting tumor immunogenicity.

**Figure 1:**
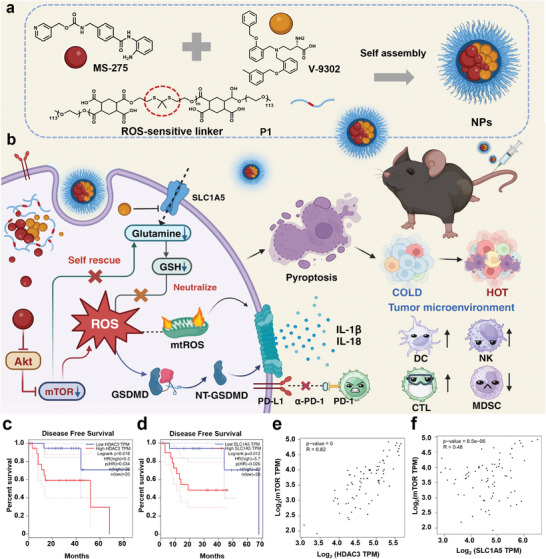
NPs induced pyroptosis in tumor cells, thereby augmenting the efficacy of tumor therapy when combined with *α*‐PD‐1. a) The strategic design of NPs to incite pyroptosis. b) NPs triggered pyroptosis, transformed “immune cold” tumors into “immune hot” tumors, thereby eliciting potent anti‐tumor immune responses in vivo. The combination of NPs and *α*‐PD‐1 further optimized tumor suppression. c) The association between disease‐free survival and HDAC3. d) The expression of SLC1A5 in patients with UVM. e) The correlation at the mRNA level between the expression of HDAC3 and mTOR in tumor tissues of UVM. f) The correlation at the mRNA level between the expression of SLC1A5 and mTOR in tumor tissues of UVM. Data in (c–f) were analyzed using GEPIA (http://gepia.cancer‐pku.cn/).

Herein, we have combined MS‐275, a commercial HDACi,^[^ ^17^ ^]^ with V‐9302, a glutamine uptake inhibitor,^[^ ^24^ ^]^ for the treatment of UVM (**Figure** **1**a). Both MS‐275 and V‐9302 show unsatisfactory pharmacokinetics, low water solubility, and poor tumor accumulation, limiting their in vivo applications.^[^ ^12^, ^25^, ^26^, ^27^ ^]^ To address these challenges, we have developed ROS‐responsive nanoparticles (NPs) loaded with both drugs. In the tumor microenvironments with high ROS levels, NPs can be degraded, releasing both MS‐275 and V‐9302, which synergistically induce the ROS storm and tumor cell pyroptosis. After intravenous injection into mice bearing tumors, NPs could selectively accumulate in the tumor sites and effectively inhibit tumor progression. When combined with programmed cell death protein 1 monoclonal antibody (*α*‐PD‐1), treatment of NPs induced a long‐lasting immune memory and prevent tumor reoccurrence (Figure 1b). Our study unlocks the potential of combining epigenetic therapies with metabolic inhibitors, offering a promising option for the treatment of UVM.

## 2 Results and Discussion

### 2.1 Expression and Correlation Analysis of HDAC and Metabolism‐Related Genes in UVM

To assess the potential of using HDACi and metabolic intervention drugs, we obtained clinical data of UVM patients from public databases and performed bioinformatics analysis. UVM patients with low expression levels of histone deacetylase (HDAC3) and the amino acid transporter SLC1A5 were found to have longer disease‐free survival (Figure 1c,d; Figure S1a,b, Supporting Information). These results suggested that the inhibition of HDAC proteins with HDACi and the inhibition of amino acid transporter with metabolic intervention drugs have the potential to treat UVM. Previous studies have reported that the perturbation of mTOR protein could be achieved by interfering with HDAC enzymes.^[^ ^17^ ^]^ SLC1A5, as a key protein in the metabolic pathway, is also associated with mTOR expression.^[^ ^28^ ^]^ Gene correlation analysis showed that the expression of HDAC3 was significantly positively correlated with mTOR (R = 0.82), whereas SLC1A5 was positively correlated with mTOR (R = 0.48). This suggested that the modulation of mTOR expression in UVM by interfering with HDAC3 may be more potent compared to that of SLC1A5 (Figure 1e,f). After focusing on mTOR expression in UVM, it was found that UVM patients with low mTOR expression had longer overall survival (Figure S1c, Supporting Information). The results of the bioinformatics analysis lend credence to our previous conjecture that it is possible to prolong the survival of UVM patients by interfering with the expression of HDAC and metabolism‐related proteins, which may have been achieved by inhibiting mTOR expression.

### 2.2 Preparation and Characterization of Nanoparticles

The bioinformatics analysis validated the potential of combining HDACi and metabolic inhibitors. Therefore, the cytotoxicity of MS‐275, V‐9302, and their combination, was first evaluated with 3‐(4,5‐dimethylthiazol‐2‐yl)‐2,5‐diphenyl‐2H‐tetrazolium bromide (MTT) assay. At an equivalent dosage, the combination treatment of MS‐275 and V‐9302 significantly outperformed each single treatment in isolation (Figure S2, Supporting Information). Inspired by the synergistic effects of MS‐275 and V‐9302, we designed NPs that encapsulated both drugs. Given the high ROS levels in the tumor environment, we built the shell of the NPs with the previously described ROS‐sensitive polymer (P1) to achieve targeted release at the tumor site.^[^ ^29^, ^30^, ^31^ ^]^

**Figure 2:**
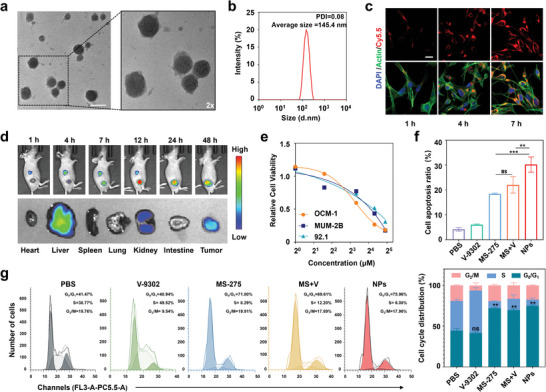
Characterization and anti‐cancer efficacy of NPs in vitro. a) TEM images of NPs. Scale bar: 100 nm. b) Hydrodynamic diameters of NPs measured by dynamic light scattering (DLS). c) CLSM images of OCM‐1 cells treated with NPs‐Cy5.5 for 1, 4, and 7 h, respectively. Scale bar: 20 µm. d) Fluorescence intensity in mice at different time points and major organs by IVIS. e) Relative cell viabilities of OCM‐1, MUM‐2B and 92.1 cells with 24 h treatment of NPs via MTT assay, respectively. f) Quantification of apoptotic ratio via flow cytometry (FCM). g) Cell cycle profiles and quantification of cell cycle ratio via FCM. All groups were treated with the following compound concentration for 24 h: MS‐275 (10 µm), V‐9302 (2 µm), MS‐275 (10 µm)+V‐9302 (2 µm) or NPs (10 µm). Data were from three independent experiments. Data are presented as mean ± SD. Statistical significance between all groups was calculated via one‐way ANOVA in (f and g). ^**^ *p* < 0. 01, ^***^ *p* < 0. 001, ns, not significant.

To prepare NPs, MS‐275 and V‐9302 were individually dissolved in DMSO and subsequently amalgamated to yield a mixture. NPs were synthesized via self‐assembly by mixing the MS‐275 and V‐9302 with P1 and PEG_2k_‐DSPE. Transmission electron microscopy (TEM) results showed that NPs had an average diameter of ≈150 nm with intact spherical structure (**Figure** **2**a). The mean particle dimensions of the NPs were 145.4 nm, with a polydispersity index (PDI) of 0.08 (Figure 2b). The size of NPs remained stable throughout 15 days of storage in serum by DLS (Figure S3a, Supporting Information). The shell of the NPs comprised the ROS‐responsive polymer P1, which, theoretically, would undergo cleavage in the high‐ROS environment of the tumor. Consequently, an in vitro simulation of a high‐ROS tumor environment was conducted. The results revealed that under high ROS conditions (10 mm H_2_O_2_), the mean particle size of the NPs was 96.3 nm, and the PDI escalated to 0.18 (Figure S3b, Supporting Information). This substantiated the capability of the NPs to undergo cleavage in a high ROS environment.

The uptake of NPs by cancer cells was examined. To visualize the cellular uptake process of NPs, they were labeled with Cy5.5 dye (red) to form NPs‐Cy5.5. OCM‐1 cells were exposed to NPs‐Cy5.5 for varying durations (1, 4, 7 h). The intensity of intracellular red fluorescence progressively amplified with the extension of treatment time, as observed through a confocal laser scanning microscope (CLSM), indicating the rapid uptake of NPs by tumor cells (Figure 2c; Figure S4, Supporting Information). To further scrutinize the biodistribution of NPs in vivo, the OCM‐1 tumor‐bearing BALB/c nude mouse model were constructed. Utilizing an in vivo imaging system (IVIS), the fluorescence signals of BALB/c nude mice intravenously injected with Cy7.5‐labeled NPs (NPs‐Cy7.5) were monitored (Figure 2d). The fluorescence intensity at the tumor site steadily escalated, peaking at ≈12 h with a value of 2.09 × 10^8^ p/s/cm^2^/sr. Over time, the fluorescence value gradually declined, with the total fluorescence value detected after 48 h being 2.00 × 10^8^ p/s/cm^2^/sr (Figure S5a, Supporting Information). Following 48 h of compound administration, ex vivo imaging of major organs and tumors in mice was conducted. The average fluorescence value of tumors ranked second among all organs at 9.72 × 10^7^ p/s/cm^2^/sr, significantly surpassing that of organs such as the heart, spleen, lungs, and intestines (Figure S5b, Supporting Information). These results indicated that NPs can rapidly accumulate in the tumor site after systemic injection, showing prolonged retention.

### 2.3 The Anti‐Cancer Activity of NPs In Vitro

To evaluate the anti‐cancer activity of NPs in vitro, the cytotoxicity of NPs in UVM cell lines (including OCM‐1, MUM‐2B, 92.1) was performed by MTT assays. Compared to the other two cell lines, the OCM‐1 cell line exhibited a lower IC_50_ of NPs (Figure 2e; Table S1, Supporting Information). The two compounds were amalgamated at the ratio present in nanoparticles as the combination group (MS+V), and the IC_50_ of MS+V could attain 6.29 µm in OCM‐1 after 48 h, which was lower than that of NPs for 24 h (Figure S6 and Table S2, Supporting Information). These results suggested that the enhanced efficacy of NPs may result from efficient cellular uptake, corroborating previous conjecture. Subsequently, cell apoptosis was analyzed under varying treatments by Annexin V‐FITC and propidium iodide (PI) double staining. Specifically, OCM‐1 cells treated with NPs exhibited an apoptosis rate of 33.3%, while the apoptosis rate of cells treated with an equivalent concentration of MS+V was 25.5% (Figure 2f; Figure S7, Supporting Information). Next, as both MS‐275 and V‐9302 demonstrated blockage of cell‐related cycles in prior studies,^[^ ^12^, ^32^, ^33^ ^]^ the effects of different compounds on the cell cycle were observed via the kit. NPs, MS‐275, and MS+V all impeded the cell cycle at the G_0_/G_1_ phase (Figure 2g). Specifically. NPs enter into the cells via endocytosis. In this way, the cellular uptake of drugs is much more efficient than molecule MS‐275 or V‐9302, while molecule V‐9302 is even insoluble in water. Additionally, NPs composed the optimized ratios of MS‐275 and V‐9302 while molecule MS‐275 or V‐9302 enter cells randomly. These findings suggested that NPs are more cytotoxic than merely combining two compounds, and that this effect was not attributable to enhancing the characteristics of a single ingredient.

### 2.4 Mechanism of Action of NPs via RNA‐seq Analysis

**Figure 3:**
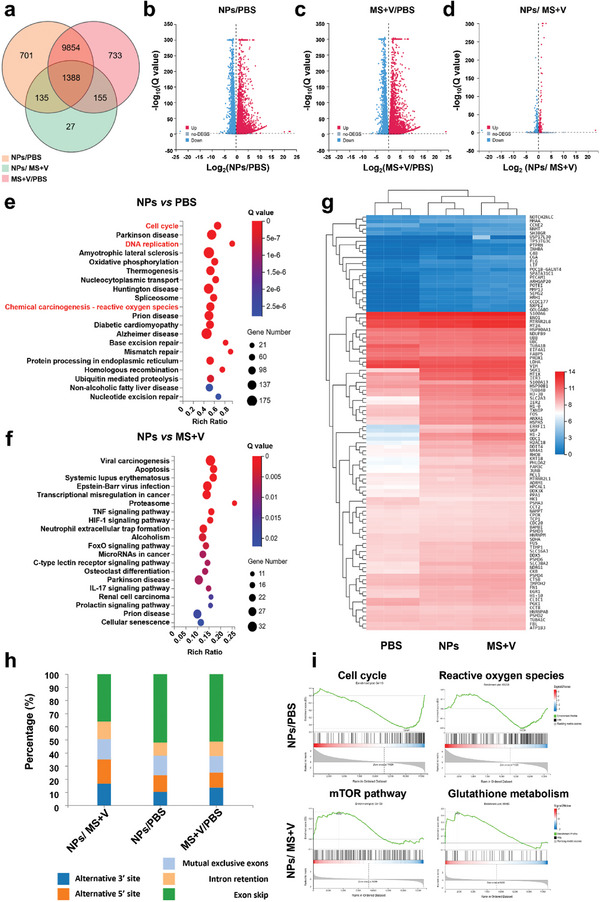
Transcription analysis of OCM‐1 cells by RNA‐seq after various treatments. a) Differential Venn diagram revealed the number of genes transcribed in each treatment group. b–d) Volcano plots displayed the differentially expressed genes. e,f) KEGG pathway enrichment analysis. g) Heatmap of gene expressions in cells treated with PBS, NPs and MS+V. h) Differential variable splicing event statistics. i) GSEA analysis showed the gene sets of Cell cycle, Reactive oxygen species, mTOR pathway and Glutathione metabolism.

To investigate the anti‐cancer mechanism of NPs and to explore the mechanism behind the enhanced efficacy post‐nanosizing, a genome‐wide RNA‐seq analysis was performed on OCM‐1 cells treated with different compounds. A total of 282185 genes were identified. 701 genes were differential expressed only in cells treated with NPs versus PBS, 733 genes were differential expressed only in cells treated with MS+V versus PBS, and 27 genes were differential expressed only in cells treated with NPs versus MS+V (**Figure** **3**a). Meanwhile, the total number of differentially expressed genes in cells treated with either NPs or MS+V was similar, suggested that the nano‐delivery system did not cause significant difference compared to simple mixed drug delivery. The 27 differentially expressed genes specific to the NPs treatment and the MS+V treatment were involved in transmembrane proteins, mTOR pathway regulation, and glutamate transport, etc (Table S3, Supporting Information). This suggested that the nanodelivery platform stimulates substance transport links such as vesicular translocation, and potentially having a stronger stimulation of the mTOR pathway and glutamate metabolism than MS+V, which was consistent with our previous results. Moreover, 6587 genes were up‐regulated (depicted as red dots) and 5491 genes were down‐regulated (depicted as blue dots) in NPs‐treated cells relative to PBS‐treated cells; 6642 genes were up‐regulated and 5488 genes were down‐regulated in MS+V ‐treated cells relative to PBS‐treated cells; 930 genes were up‐regulated and 775 genes were down‐regulated in NPs‐treated cells relative to MS+V‐treated cells (Figure 3b‐d). After treatment, more differential genes showed a tendency to be up‐regulated. The genes differentially expressed in the NPs and the PBS were then subjected to Kyoto Encyclopedia of Genes and Genomes (KEGG) enrichment analysis. NPs primarily impacted the pathways involved in the Cell cycle, DNA replication, and Chemical carcinogenesis‐reactive oxygen species (Figure 3e). Moreover, NPs significantly affected the expression of genes in multiple signaling pathways of differentially expressed genes in the NPs versus MS+V (Figure 3f). The preceding results indicated that NPs interfered with Cell cycle, DNA replication, and Chemical carcinogenesis‐reactive oxygen species; and the effect of nanosizing on anti‐cancer efficacy was primarily achieved by influencing various signaling pathways.

To further explore the differences between the groups, the differentially expressed genes after treatment in each group were visualized by a heatmap (Figure 3g). Compared to PBS, cells treated with the two compounds exhibited an upsurge in the expression of some pivotal genes, such as ERRFI1 and H1‐2, which potentially inhibit tumor invasion and metastasis and influence the function of downstream histone proteins. The differences between NPs and MS+V were relatively modest. Consequently, alternative splicing analysis was employed to probe the causes of the variations between NPs and MS+V (Figure 3h). Alternative splicing is the process of generating diverse mRNA splicing isoforms from an mRNA precursor via different splicing methods (selecting varying combinations of splice sites).^[^ ^34^ ^]^ It is a crucial mechanism for regulating gene expression and engendering proteomic diversity. The ratio of exon skip underwent significant alteration between NPs and MS+V, indicating that the performance difference between the two may be reflected through exon skip. Subsequently, according to Gene Set Enrichment Analysis (GSEA) analysis, NPs considerably down‐regulated the expression of genes related to cell cycle in OCM‐1 cells, whereas the expression of genes associated with reactive oxygen species was moderately changed. RNA expression in the mTOR pathway and glutathione metabolism was dramatically escalated by NPs as compared to MS+V (Figure 3i). These results collectively prove that NPs possess enhanced anti‐cancer properties by simultaneously influencing the mTOR pathway and glutamine metabolism.

### 2.5 NPs Formed a ROS Storm and Induced Pyroptosis In Vitro

**Figure 4:**
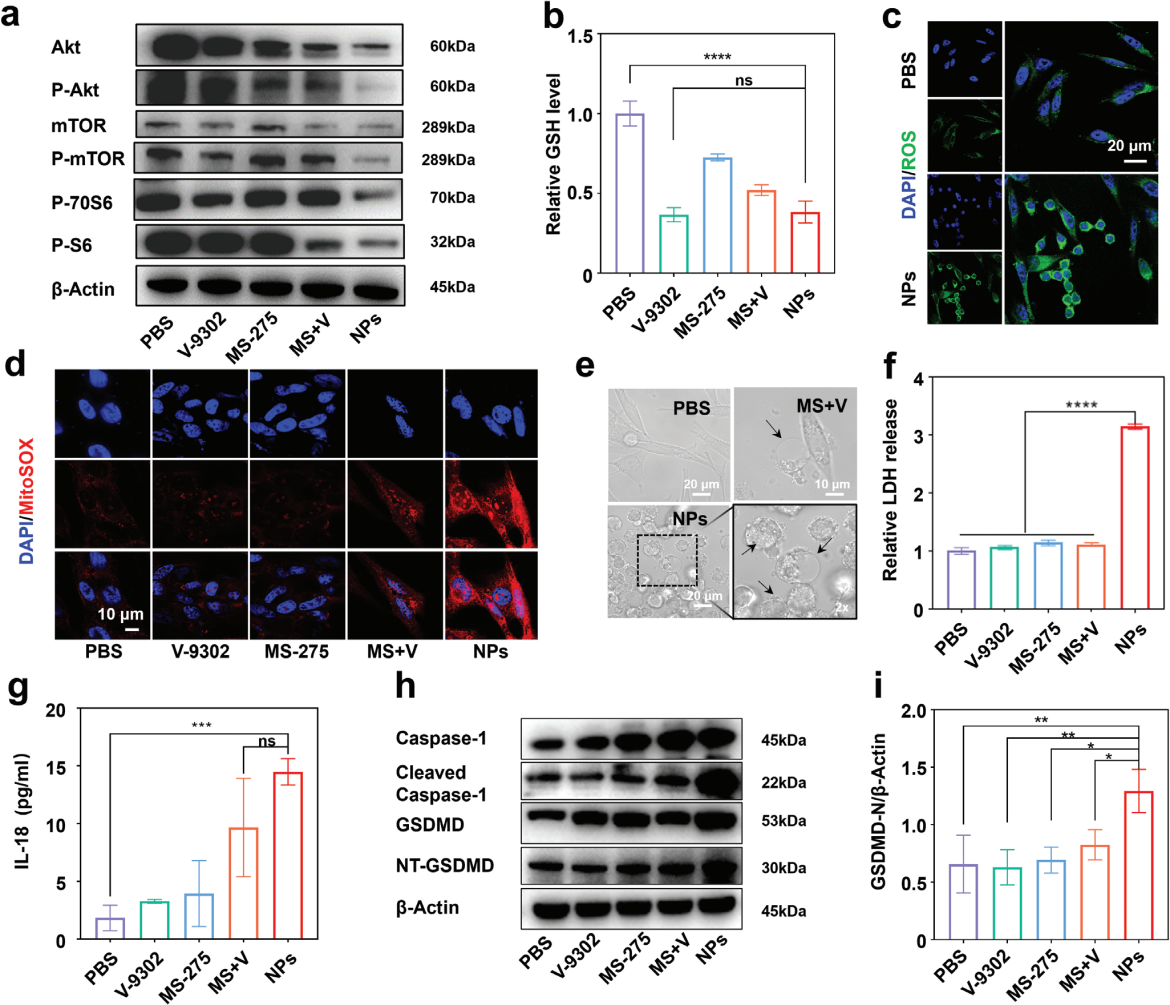
NPs interfered with the mTOR pathway and deprivated glutamine to form ROS storms, which induced pyroptosis in vitro. a) Expression levels of mTOR proteins related in OCM‐1 cells after various treatments for 24 h by WB. *β*‐Actin was used as the internal reference protein. b) Relative GSH level of cells after various treatments. c) CLSM images of ROS in cells after various treatments. (blue, DAPI; green, ROS). d) CLSM images of mitoROS in cells. (blue, DAPI; red, MitoSOX). e) Pyroptosis cells under microscope treated with PBS, MS+V and NPs for 24 h. f) Relative LDH level in OCM‐1 cells supernatant of different groups. g) Quantification of IL‐18 in OCM‐1 cells supernatant of different groups. h) Expression levels of pyroptosis‐related proteins in cells after various treatments for 24 h by WB. *β*‐Actin was used as the internal reference protein. i) Relative expression of NT‐GSDMD. Data were from three independent experiments. Data were presented as mean ± SD. Statistical significance between all groups was calculated via one‐way ANOVA in (b, f, g, and i). ^*^ *p* < 0.05, ^**^ *p* < 0. 01, ^***^ *p* < 0.001, ^****^ *p* < 0.0001, ns, not significant.

Previous genome‐wide RNA‐seq results suggested that the efficacy of NPs may be achieved by interfering with the mTOR pathway and glutathione metabolism, aligning with the characteristics of the two individual compounds. To detect the perturbation of the mTOR pathway instigated by NPs, the expression of pertinent proteins in cells post‐treatment was observed via western blotting (WB). The expression of Akt, Phospho‐Akt, mTOR, Phospho‐mTOR, and downstream proteins were down‐regulated in cells treated with NPs and MS+V (**Figure** **4**a; Figure S8, Supporting Information). Specifically, MS‐275 reduced the expression of Akt, and V‐9302 had a greater effect on the expression of Phospho‐mTOR. Relevant proteins in the mTOR pathway such as Akt, Phospho‐Akt, Phospho‐S6 were down‐regulated to a certain extent after treated with MS+V. NPs encapsulated both MS‐275 and V‐9302, which inherited the properties of the single compound in previous experiments. The effects of MS+V can even be amplified due to the efficient delivery capabilities of nanosystems. As a result, the relevant proteins of the mTOR pathway were significantly down‐regulated in the cells treated with NPs. Subsequently, the level of GSH was assessed, and the results indicated that NPs have the same strength as V‐9302 to reduce GSH content in cellular (Figure 4b). NPs effectively suppressed the mTOR pathway and led to a decrease in intracellular GSH levels, which could increase ROS production, and decrease ROS consumption.^[^ ^35^, ^36^ ^]^ Therefore, NPs may cause a lethal ROS storm.

To observe the ROS storm more precisely, ROS probes were employed to detect intracellular ROS (green) via CLSM (Figure 4c; Figure S9a, Supporting Information). It was observed that NPs induced a substantial amount of intracellular ROS production. The ROS generation was further quantified by FCM. Specifically, the ROS generated by NPs were 17‐fold and 1.7‐fold higher than that of PBS and MS+V, respectively (Figure S9b,c, Supporting Information). Intracellularly, one of the major sources of ROS is the substrate end of the respiratory chain in the inner mitochondrial membrane. To explore the source of ROS induced by intervention, MitoSOX was used to specifically bind with mitochondrial ROS (red). The results demonstrated that NPs indeed induced a substantial release of mitochondrial ROS (Figure 4d). The above results suggested that NPs create an intracellular ROS storm in cells by simultaneously inhibiting the mTOR pathway and reducing GSH production. Meanwhile, mitochondrial ROS accounted for a proportion of ROS storm, which favored the formation of cellular pores during pyroptosis.^[^ ^21^ ^]^

The ROS storm set the stage for pyroptosis. A distinct pyroptosis phenomenon was observed under the microscope (Figure 4e). The black arrows indicated the vesicular structures formed by the flow of contents out of the cell membrane after perforation when pyroptosis occurred. Following treatment with different compounds for 24 h, supernatant medium was collected to detect lactate dehydrogenase (LDH) and adenosine 5′‐triphosphate (ATP), indicating cell rupture. It was observed that NPs significantly elevated LDH and ATP levels in the cell supernatant (Figure 4f; Figure S10a, Supporting Information). As cells undergo pyroptosis, they release interleukin‐1*β* (IL‐1*β*) and interleukin‐18 (IL‐18), recruiting additional inflammatory cells and amplifying the inflammatory response.^[^ ^19^ ^]^ IL‐18 and IL‐1*β* levels in supernatant media were detected by an Elisa kit. Specifically, the levels of IL‐18 and IL‐1*β* in the supernatant of NPs treated cells were 14.48 and 41.53 pg mL^−1^, respectively, which were 7.87 times and 1.60 times higher than the corresponding cytokines in untreated cells (Figure 4g; Figure S10b, Supporting Information). Subsequently, the results of WB were performed to evaluate protein expression of the pyroptosis‐related pathway. The results revealed that NPs significantly upregulated the expression of NT‐GSDMD and cleaved caspase‐1 (Figure 4h). The expression of NT‐GSDMD generated by NPs was 1.97 times higher than that of the PBS, as indicated by the normalized statistics of the internal reference protein *β*‐Actin (Figure 4i; Figure S11, Supporting Information). These findings suggested that NPs could induce a ROS storm and trigger pyroptosis within cancer cells.

### 2.6 Anti‐Tumor Effects of NPs in a BALB/C Nude Mouse Tumor Model

**Figure 5:**
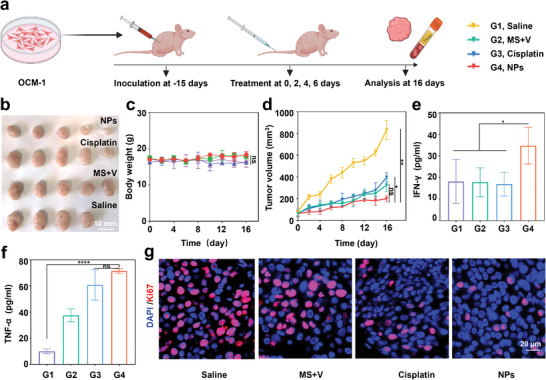
In vivo anti‐tumor effects of the NPs. a) Schematic illustration of in vivo treatment. b) Representative tumor image of different groups after 16 days of treatment. c) Body weight changes and d) tumor growth inhibition curves of mice treated with saline, MS+V, cisplatin and NPs at 4 mg kg^−1^ body weight. n = 5 mice per group. e) IFN‐*γ* level in serum of mice with various treatments. f) TNF‐*α* level in serum of mice with various treatments. g) Immunofluorescence imaging of the expression of Ki67 in OCM‐1 tumor after different treatment. Data are presented as mean ± SD. Statistical significance between all groups was calculated via two‐way ANOVA in (c and d), one‐way ANOVA in (e and f). ^*^ *p* < 0.05, ^**^ *p* < 0.01,^****^ *p* < 0.0001, ns, not significant.

The anti‐tumor efficacy of NPs was then examined in a BALB/C nude mouse model based on OCM‐1 cells (**Figure** **5**a). Fifteen days after tumor engraftment, animals were treated with saline, MS+V, cisplatin, or NPs. Considering that cisplatin is incorporated in the initial chemotherapy regimen for UVM, the administered dose of cisplatin was referenced.^[^ ^37^, ^38^ ^]^ Over the course of the 16‐day treatment cycle, it was observed that the subcutaneous tumors in mice treated with saline were larger in size, whereas the tumors in all other groups that received the intervention were smaller (Figure 5b). There was no significant change in the weight of the animals during the course of treatment (Figure 5c). The measured tumor volumes of the mice were analyzed, and the results demonstrated that the tumors in the mice treated with cisplatin increased by 330.72 mm^3^, while the tumors in the mice treated with NPs increased by 137.94 mm^3^, with the former being 2.40 times larger than the latter. In contrast, the tumors in the mice after receiving a simple combination of the two individual compounds increased by 260.93 mm^3^, which was 1.89 times larger than the tumors in the mice treated with NPs (Figure 5d). Statistical analysis revealed no significant difference between NPs and MS+V groups, but significant differences were observed between NPs versus Cisplatin (*P* < 0.05) and NPs versus Saline groups (*P* < 0.01). Concurrently, H&E staining was performed on major organs (heart, liver, spleen, lungs, and kidneys) of mice under a fluorescence microscope. The major organs of mice treated with NPs did not exhibit any noticeable morphological changes compared to the histomorphology of mice treated with saline, further attesting to the sufficient safety of NPs (Figure S12, Supporting Information). Collectively, these findings suggested that NPs exhibit low toxicity and high efficiency.

The expression of interferon (IFN)‐*γ* and tumor necrosis factor (TNF)‐*α* in the serum of mice were evaluated by ELISA (Figure 5e,f). Following treatment with NPs, IFN‐*γ* in the serum of mice escalated to 34.82 pg mL^−1^, while TNF‐*α* increased to 71.45 pg mL^−1^, both of which were significantly up‐regulated compared to the mice treated with saline. This suggested that NPs could instigate a systemic response. To observe the proliferation of tumor cells, the mouse tumor tissues were excised to stain them with Ki67 immunofluorescence. Ki‐67 protein is a nuclear protein associated with the cell cycle. Cells in the growth and proliferation phase will be labeled as Ki‐67 positive expression.^[^ ^37^ ^]^ The proliferative capability of the tumor cells was likely inhibited post‐treatment with NPs, as evidenced by the significant reduction in Ki67‐positive cells in treated with NPs and the abundance of Ki67‐positive cells in treated with saline (Figure 5g). In situ Ki67 assays suggested that the fluorescence in the PBS‐treated mice was 3.29‐fold stronger than that in the NPs‐treated mice (Figure S13, Supporting Information). The findings indicated that NPs can successfully halt tumor growth and may potentially enhance the body's response against tumors.

### 2.7 NPs Elicited Anti‐Tumor Immune Responses and Long‐Term Immune Memory In Vivo

**Figure 6:**
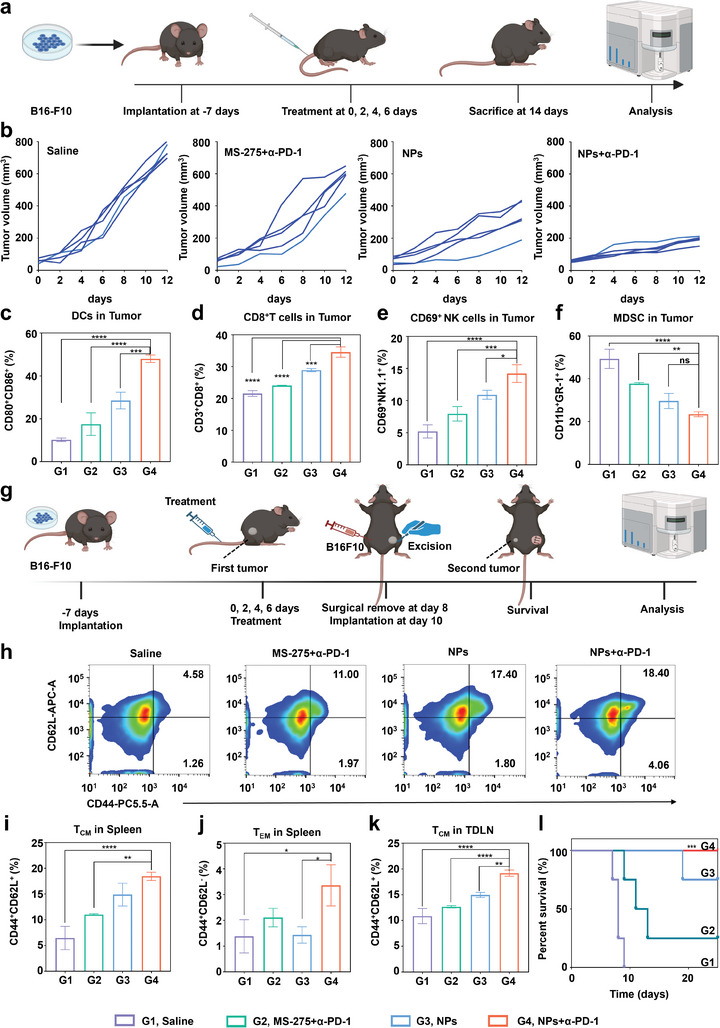
NPs induced an anti‐tumor immune response and enhanced immune memory in combination with *α*‐PD‐1 in vivo. a) Schematic illustration of treatment schedules in vivo. b) Tumor growth inhibition of mice treated with various compounds. n = 5 mice per group. c) The percentages of DC cells (CD80^+^CD86^+^) within tumor in each group are presented as histograms. d) The percentages of CD8^+^ T cells (CD3^+^CD8^+^) within tumor in each group are presented as histograms. e) The percentages of populations of NK cells (CD3^−^NK1.1^+^CD69^+^KLRG1^+^) within tumor in each group are presented as histograms. f) The percentages of populations of MDSCs (CD11b^+^GR‐1^+^) within tumor in each group are presented as histograms. g) Schematic illustration of treatment schedules in vivo. h) Representative FCM analysis images and i) populations of T_CM_ (CD44^+^CD62L^+^) in spleen. j) The percentages of populations of T_EM_ (CD44^+^CD62L^−^) in spleen. k) The percentages of populations of T_CM_ (CD44^+^CD62L^+^) in TDLNs. l) Survival analysis of mice tumor up to day 25. Data are presented as mean ± SD. Statistical significance between all groups was calculated via one‐way ANOVA in (c–f, i–k), and unpaired two‐sided t‐test in l). ^*^ *p* < 0.05, ^**^ *p* < 0.01, ^***^ *p* < 0.001, ^****^ *p* < 0.0001. ns, not significant.

Previous findings suggested that NPs induced tumor cell pyroptosis, promoted the secretion of IL‐1*β* and IL‐18, and increased the serum levels of IFN‐*γ* and TNF‐*α* in mice. These cytokines can trigger positive immunological regulation when they stimulate the immune system. Meanwhile, the administration of NPs enhanced PD‐L1 expression in vitro (Figure S14, Supporting Information). Previous studies have shown that the level of PD‐L1 expression in tumor tissues can serve as an indicator of the efficacy of PD‐L1/PD‐1 monoclonal antibody.^[^ ^38^ ^]^ Researchers combined MS‐275 with *α*‐PD‐1 for the treatment of metastatic UVM in the reported PEMDAC phase 2 clinical trial, achieving long‐lasting therapeutic results.^[^ ^39^ ^]^ Consequently, a subcutaneous tumor model in the B16‐F10 cell line was established for group therapy to investigate whether NPs could induce an anti‐tumor immune response in vivo and enhance the therapeutic response rate of *α*‐PD‐1 (**Figure** **6**a). After monitoring tumor growth to ≈80 mm^3^, five mice were randomly assigned per group to four different groups: Saline, MS‐275 (4 mg kg^−1^ intravenously)+*α*‐PD‐1 (10 mg kg^−1^ intraperitoneally), NPs (4 mg kg^−1^ intravenously), and NPs (4 mg kg^−1^ intravenously)+*α*‐PD‐1 (10 mg kg^−1^ intraperitoneally). The body weights and tumor volumes of the mice were measured and recorded every other day starting from the day of treatment. The tumor volume recording curves in mice revealed that NPs+*α*‐PD‐1 had the most potent anti‐tumor efficacy (Figure 6b; Figure S15, Supporting Information). Subsequently, tumors and tumor draining lymph nodes (TDLNs) from different groups were procured, and the relevant immunological indicators were correlated to further investigate the impact of NPs on the tumor immune microenvironment. Dendritic cells (DC cells) act as sentinels of the immune system and are able to rapidly ingest antigens and process them for presentation to immature T cells.^[^ ^40^ ^]^ Compared to saline, NPs+*α*‐PD‐1 dramatically increased the proportion of mature DC cells in TDLNs and tumors where the population of CD80^+^CD86^+^ cells was significantly increased from 13.83% to 41.83%, and from 10.24% to 47.97%, respectively (Figure 6c; Figures S16 and S17, Supporting Information). Cytotoxic T lymphocytes (CTL) are a crucial type of immunological surveillance cell.^[^ ^41^, ^42^ ^]^ Higher percentages of CTL in patients tumors can help suppress tumor progression and even eliminate the tumor over long periods.^[^ ^43^ ^]^ Therefore, high percentages of CTL with cytotoxic function in tumor tissues is a positive prognostic indicator. The infiltration of CD8^+^ T lymphocytes in the tumor tissues of groups were examined using FCM (Figure 6d; Figure S18, Supporting Information). The percentage of CD8^+^ T cells (CD3^+^ CD8^+^) in the tumor tissues of mice given NPs+*α*‐PD‐1 was ≈1.4 times more than that of the mice given MS‐275+*α*‐PD‐1. This suggested that coadministration of NPs and *α*‐PD‐1 may be more beneficial for cytotoxic T cells to function.

Natural killer cells (NK cells), constitute the third major class of lymphocytes following T cells and B cells. They are the primary source of IFN‐*γ* release and possess the capability to recognize and attack viruses, tumor cells, and foreign bodies.^[^ ^44^ ^]^ NK cells were concerned in tumors since previous findings indicated an elevation in IFN‐*γ* expression in mice treated with NPs. Specifically, the percentage of NK cells following NPs+*α*‐PD‐1 therapy could reach 14.20%, which is nearly triple as high as the Saline. The proportions of NK cells in the NPs and MS‐275+*α*‐PD‐1 were 10.90% and 7.94%, respectively (Figure 6e; Figure S19, Supporting Information). Such a trend suggested that the tumor may be more reliant on NPs to induce cellular pyroptosis, followed by NK cell aggregation. Myeloid‐derived suppressor cells (MDSCs) can inhibit the normal innate and adaptive immunological functions of the body's immune cells.^[^ ^45^ ^]^ Therefore, the proportion of MDSCs was assessed in tumor tissues (Figure 6f; Figure S20, Supporting Information). Following treatment with NPs+*α*‐PD‐1, the proportion of MDSCs in tumor tissues was 23.49%, which was ≈47.65% of the Saline. These results suggested that the combination of NPs and *α*‐PD‐1 could effectively increase NK cells infiltration and mitigate the original immunosuppression in the tumor.

In the immune responses against malignancies, T cells and other immune cells can become exhausted.^[^ ^46^ ^]^ The long‐term effects of NPs in conjunction with *α*‐PD‐1 were investigated in C57BL/6 mice model (Figure 6g). Two million B16‐F10 cells were injected into the right buttock subcutaneously of C57BL/6 mice. Four treatments were initiated once the tumor volume reached ≈80 mm^3^. Two days after the completion of the fourth treatment, the tumor was surgically excised. After a two‐day rest period, an equal number of B16‐F10 cells were injected into the mice's left buttock subcutaneously, allowing for the tracking of the development of the second tumor. A portion of the mice in each group were sacrificed once their second tumor volume reached 500 mm^3^, and their spleens and TDLNs were removed to monitor the ratio of memory T cells (T_CM_, CD3^+^CD8^+^CD44^+^CD62L^+^) and effector T cells (T_EM_, CD3^+^CD8^+^CD44^+^CD62L^−^). Following NPs+*α*‐PD‐1, the percentage of T_CM_ in the spleen increased to 18.47%, which is approximately three times higher than in those treated with saline (Figure 6h,i; Figure S21, Supporting Information). In mice treated with NPs+*α*‐PD‐1, the percentage of T_EM_ in spleen was 3.36% (Figure 6j). After receiving NPs+*α*‐PD‐1, the percentage of T_CM_ in TDLNs was 19.20%, while it was only 10.86% in those treated with saline (Figure 6k; Figure S22, Supporting Information). The development of the second tumor volume to 100 mm^3^, which was recorded as state 1, was used to plot survival curves (Figure 6l). Mice treated with NPs+*α*‐PD‐1 did not achieve state 1 over the 25‐day observation period, and some did not even develop a second tumor. The findings described above suggested that NPs and *α*‐PD‐1 together help to improve mice's immunological memory and prevent tumor recurrence.

## 3 Conclusion

In conclusion, we have developed an innovative ROS‐responsive nano‐delivery system (NPs), which co‐delivers a HDACi, MS‐275, and a metabolic inhibitor, V‐9302, for the treatment of UVM. This targeted approach exploits the enhanced permeability and etention effect, ensuring that our NPs accumulate at the tumor site. Upon arrival, these NPs respond to the high ROS levels within the tumor microenvironment, releasing their therapeutic payload. In mouse melanoma models, NPs effectively suppressed tumor progression and improved anti‐tumor immunity by recruiting DC cells, CD8^+^ T cells, and NK cells, converting the “immune‐cold” tumor to “immune‐hot” tumor. Furthermore, when combined with *α*‐PD‐1, NPs effectively promote immunological memory and prevent tumor reoccurrence. By enhancing immunogenicity and immunotherapy responsiveness through combining HDACi with metabolic inhibitors, our study lays a groundwork for future clinical applications and provides a potential blueprint for combatting tumor resistance.

## Conflict of Interest

The authors declare no conflict of interest.

## Supporting information

Supporting Information

## Data Availability

The data that support the findings of this study are available from the corresponding author upon reasonable request.
