# Supplementary material for: Co‐delivery Nano System of MS‐275 and V‐9302 Induces Pyroptosis and Enhances Anti‐Tumor Immunity Against Uveal Melanoma
Source: Adv Sci (Weinh). 2024 Jun 18;11(31):2404375. doi: 10.1002/advs.202404375 (PMC11336933; doi:10.1002/advs.202404375)
Supplement: Supplementary file 1 — Supporting Information [file ADVS-11-2404375-s001.pdf]

ADVANCED SCIENCE
Open Access

Supporting Information

for *Adv. Sci.*, DOI 10.1002/advs.202404375

Co-delivery Nano System of MS-275 and V-9302 Induces Pyroptosis and Enhances Anti-Tumor Immunity Against Uveal Melanoma

*Hong Ren, Zhenkai Wu, Jia Tan, Hui Tao, Wangyuan Zou, Zheng Cao, Binyu Wen, Ziyi Cai, Jiaqi Du and Zhihong Deng**

Supporting Information

Co-delivery Nano System of MS-275 and V-9302 Induces Pyroptosis and Enhances Anti-Tumor Immunity Against Uveal Melanoma

*Hong Ren, Zhenkai Wu, Jia Tan, Hui Tao, Wangyuan Zou, Zheng Cao, Binyu Wen, Ziyi Cai, Jiaqi Du, and Zhihong Deng**

SUPPLEMENTAL MATERIALS AND METHODS

1. Materials

All chemicals were obtained from commercial sources and used without further purification unless otherwise noted. All reagents were used as received unless otherwise specified.

MS-275 were purchased from MCE (Shanghai, China). V-9302, 3-(4,5-dimethylthiazol-2-yl)-2,5-diphenyltetrazolium bromide (MTT) and sodium dodecyl sulfate (SDS), PEG_{2k}- DSPE were purchased from Aladdin Co. Ltd (Shanghai, China). Data related to ROS-sensitive polymer P1 were extracted directly from our previous study[1].

Cell culture vessels were purchased from Corning (Corning, NY, USA). DMEM medium, RPMI 1640 medium, fetal bovine serum (FBS), 0.25% trypsin-EDTA, and penicillin/streptomycin (P/S) were purchased from (Gran Island, NY, USA). 2-(4-amidinophenyl)-1H-indole-6-carboxamidine (DAPI), propidium iodide (PI) and FITC Phalloidin were purchased from Solarbio Science & Technology *Co.,Ltd.* (Beijing, China). Annexin V-FITC/PI Cell Apoptosis Kit, Calcein/PI Live/Dead Viability/Cytotoxicity Assay Kit, bicinchoninic acid (BCA) protein assay kit and TUNEL Apoptosis Assay Kit were purchased from Beyotime (Shanghai, China).

Goat Anti-Rabbit IgG H&L (Alexa Fluor® 488) (ab150077) and Goat Anti-Rabbit

IgG H&L (Alexa Fluor® 555) (ab150078) were purchased from Abcam.

Cleaved Caspase-1 (Asp297) (D57A2) Rabbit mAb #4199, Gasdermin D (E9S1X) Rabbit mAb #39754, Cleaved Gasdermin D (Asp275) (E7H9G) Rabbit mAb #36425, Akt Antibody #9272, Phospho-Akt (Ser473) (D9E) XP® Rabbit mAb #4060, mTOR Antibody #2972, Phospho-mTOR (Ser2448) Antibody #2971, p70 S6 Kinase (E8K6T) XP® Rabbit mAb #34475, Phospho-S6 Ribosomal Protein (Ser240/244) (D68F8) XP® Rabbit mAb #5364 were purchased from Cell Signaling Technology. Caspase-1 Rabbit Monoclonal Antibody (AF1681), β-Actin Rabbit Monoclonal Antibody (AF5003) was purchased from Beyotime (Shanghai, China).

PE anti-mouse CD11c antibody, FITC anti-mouse CD80 antibody, APC anti-mouse CD86 antibody, PE anti-mouse CD3 antibody, FITC anti-mouse CD8 antibody, APC anti-mouse CD4 antibody, APC anti-mouse CD62L antibody, PerCP/Cyanine5.5 anti-mouse CD44 antibody, PerCP/Cyanine5.5 anti-mouse CD45 antibody, APC anti-mouse $CD11b^+$ antibody, FITC anti-mouse $GR\text{-}1^+$ antibody, PerCP/Cyanine5.5 anti-mouse CD3 antibody, PE anti-mouse NK 1.1 antibody, APC anti-mouse CD69 antibody and FITC anti-mouse $KLRG1^+$ antibody were purchased from Biolegend, USA.

2. Instrumentation and methods:

The size distribution of nanoparticles was measured by dynamic light scattering (DLS, Malvern Zetasizer, UK). The morphology and size of nanoparticles were characterized by transmission electron microscope (TEM, Hitachi HT7700, Japan). Localization of nanoparticles and all the immunofluorescence slides were imaged using a confocal laser scanning microscope (CLSM, LSM 800, ZEISS, Germany). MTT assay was conducted using a microplate reader (SpectraMax, USA). *In vivo* imaging was conducted by *In Vivo* Imaging System (IVIS, Perkin Elmer, USA). Fluorescence intensity in cells was carried out using a CytoFLEX Flow Cytometry (Beckman Coulter, USA).

3. Cell Culture

OCM-1, MUM-2B, 92.1 and B16-F10 cells were used in *in vitro* and *in vivo* experiments. OCM-1, MUM-2B and 92.1 was cultured in DMEM media. B16-F10 cells were cultured in RPMI 1640 media. Culture mediums were supplemented with 10% (v/v) FBS, 1% (v/v) P/S. All the cell lines were cultured in an incubator at 37 °C containing 5% (v/v) CO_2.

4. Preparation of NPs, NPs-Cy5.5, NPs-Cy7.5

MS-275 (10 mg), V-9302 (10 mg), P1 (40 mg) and PEG_{2k}-DSPE (30 mg) was dissolved in DMSO (1 mL), and the mixture was then added dropwise into water (10 mL) under continuous agitation. The suspension was then dialyzed against water (molecular cutoff Mw=3500) to remove DMSO. Finally, NPs was obtained by collecting supernatant after centrifugation separation (3000 rpm, 5 mins). The concentration of NPs was determined by High Performance Liquid Chromatography (HPLC). Additional Cy5.5/ Cy7.5 dye was added when preparing NPs-Cy5.5/ Cy7.5 and the rest of the steps were as above.

5. *In vitro* cellular uptake of NPs by CLSM and flow cytometry (FCM)

A cover slide was placed in the bottom of each well of a 24-well plate. Cells (1×10^5) in 1 mL medium were added to each well and incubated at 37 °C for 12 h. Then, the cells were incubated with NPs-Cy5.5 diluted in cell media for various time intervals (1 h, 4 h, 7 h). Next, the cells were washed with phosphate-buffered saline (PBS) and further incubated with the nucleus specific stain DAPI (ab285390, Abcam) and the cytoskeleton specific stain Alexa-488 (1:500, Beyotime) for 0.5 h. Subsequently, the cellular uptake was assessed by CLSM (DAPI, λ_{ex} = 405 nm, λ_{em} = 460 nm, Cy5.5, λ_{ex} = 673 nm, λ_{em} = 692 nm). The Zen software was used to analysis and draw the pictures.

To perform flow cytometry, cells were seeded into 12-well plate at 3×10^5 cells/well and incubated at 37 °C for 12 h. The NPs-Cy5.5 were added to each well in different time points (1 h, 4 h, 7 h), and the wells without any treatment were performed as negative control. Afterwards, the cells were harvested and quantified using FCM. The

flowjo software was used to analysis.

6. *In vitro* cytotoxicity study

MTT assay was used to examine the cytotoxicity. Cells were seeded into 96-well plates (8×10^3 cells/well) and then incubated at 37 °C overnight. Then, the cells were treated with MS-275, V-9302, MS-275+V-9302, NPs for 24/48 h, respectively. MTT reagent in DMEM (10 μL) was added into each well and allowed to incubate for 4 h. 10% SDS (100 μL) was added to replace the media. After gentle agitation (5 mins), the absorbance of each well was recorded on a microplate reader (Spectra Max) at 570 nm (peak absorbance) and 650 nm (peak background).

7. Cell apoptosis assays

OCM-1 cells were seeded in 12-well plates (3×10^5 cells /well) and incubated with MS-275(10 μM), V-9302 (2 μM), MS-275(10 μM) + V-9302 (2 μM), NPs (10 μM). After 24 h, apoptotic cells were detected by flow cytometry (Becton Dickinson and Company, USA), using Annexin V-FITC/PI Cell Apoptosis Kit (Beyotime, C1062L).

8. Cell Cycle

OCM-1 cells were seeded in 12-well plates (3×10^5 cells /well) and incubated with MS-275(10 μM), V-9302 (2 μM), MS-275(10 μM) + V-9302 (2 μM), NPs (10 μM). After 24 h, cells were detected by flow cytometry (Becton Dickinson and Company, USA), using Cell Cycle and Apoptosis Analysis Kit (Beyotime, C1052).

9. Intracellular ROS generation

A cover slide was placed in the bottom of each well of a 24-well plate. Cells (1×10^5) in 1 mL complete media were added to each well and incubated at 37 °C for 12 h. Afterward, the cells were treated with MS-275(10 μM), V-9302 (2 μM), MS-275(10 μM) + V-9302 (2 μM), NPs (10 μM) at the photosensitive unit for 7 h respectively. Subsequently, the culture medium of the cells was replaced with a serum-free medium

and then incubated with ROS indicator DCFH-DA (10 μM) for 20 mins. Then the cover slide of each well was placed on the microslide, and the cell nuclei were stained with DAPI. Subsequently, images were collected with CLSM.

Furthermore, the intracellular ROS level was further detective and quantify by FCM. First, cells were seeded in 12-well plate at a density of 3 $\times 10^5$ per well and incubated at 37 °C for 12 h. Afterward, the cells were treated with the same conditions as the above CLSM analysis. Finally, the cells were harvested to examine the intracellular DCF by FCM.

10. *In vitro* fluorescence staining

OCM-1 cells were seeded on 24-well plates (1×10^4 cells/well) and allowed to adhere overnight. The cells were then treated with MS-275(10 μM), V-9302 (2 μM), MS-275(10 μM) + V-9302 (2 μM), NPs (10 μM) for 24 h; PBS served as control treatment.

PD-L1: The above cells were washed with PBS and further incubated with PD-L1 Rabbit Polyclonal Antibody for 2 h at 37 °C. Subsequently, the cells were incubated with Goat Anti-Rabbit IgG H&L (Alexa Fluor® 555) for 1 h at 37 °C. The expression of PD-L1 inside the cells was observed by CLSM.

MitoSox: The above cells were washed with PBS and further incubated with MitoSox for 2 h at 37 °C. Then the cover slide of each well was placed on the microslide, and the cell nuclei were stained with DAPI. Subsequently, images were collected with CLSM.

11. GSH and GSSG Assay Kit, ATP Assay Kit, LDH Assay Kit

OCM-1 cells were seeded in 12-well plates (3×10^5 cells /well) and incubated with MS-275(10 μM), V-9302 (2 μM), MS-275(10 μM) + V-9302 (2 μM), NPs (10 μM) for 24 h. Cells were detected using GSH and GSSG Assay Kit, cellular supernatant was detected using ATP Assay Kit (Beyotime, S0027) and LDH Assay Kit (Beyotime,

C0016).

12. Western blot

OCM-1 cells were seeded in 6-well plates (6×10^5 cells/well) and allowed to adhere overnight. The cells were treated with MS-275(10 μM), V-9302 (2 μM), MS-275(10 μM) + V-9302 (2 μM), NPs (10 μM) for 24 h. Cells were washed three times with cold PBS and maintained in medium for 0.5 h. RIPA lysis buffer with protease and phosphatase inhibitors was added into well. The proteins of cells were extracted through centrifuge at a speed of 12000 rpm for 5 mins. Protein content quantification was carried out by the BCA protein assay kit (Beyotime, P0011). Then, the electrophoreses process was conducted through SDS-PAGE by a gel-electrophoretic apparatus (Bio-Rad mini, USA), and the proteins were transferred to the PVDF films and incubated with the antibodies against various proteins overnight on a shaker at 4°C. Subsequently, the PVDF films were washed 5 times and incubated with HRP conjugated antibodies for 1 h. The Western blot images were obtained by Amersham Imager 600 (AI600, General Electric *Co., Ltd.,* USA) with 300 μL of ECL chemiluminescent reagent (Beyotime biotechnology *Co., Ltd.*, P0018AS) added on the top of the membrane.

13. *In vivo* biodistribution analysis

The biodistribution of nanoparticles was investigated by *in vivo* imaging system (IVIS, Spectrum CT， PerkinElmer). OCM-1 cells (5×10^6 cells) were injected into right buttock subcutaneously of BALB/c nude mice. When the tumor volumes reached about 80 mm^3, the mice were injected with NPs-Cy7.5 intravenously. After injection, the fluorescence imaging was performed by IVIS spectrum imaging system (Ex/Em=745 nm/840 nm) at various time points. Mice were sacrificed at 48 h post injection for fluorescence imaging of the major organs (heart, liver, spleen, lung, intestine, and kidney) and tumor tissues *ex vivo.*

14. Establishment subcutaneous tumor model of OCM-1 and therapeutic effect evaluation

To investigate the antitumor effect of nanoparticles, OCM-1 cells (5×10^6 cells) were injected into right buttock subcutaneously of BALB/c nude mice. When the tumor volumes reached about 80 mm^3, the mice were injected with Saline, Cisplatin, MS-275+V-9302, and NPs at the dose of 4 mg/kg intravenously. The tumor volume was monitored at a time interval of 2 days. Tumor volume was calculated by the following formula:

$$\text{Volume} = (\text{Length} * \text{Width}^2) / 2.$$

15. Immune response analysis *in vivo*

To examine immune response *in vivo*, B16-F10 cells (2×10^6 cells) were injected into right buttock subcutaneously of C57BL/6 mice, the mice bearing the subcutaneous tumor model of B16-F10 were treated with various formulations. The tumors, tumor draining lymph nodes (TDLNs) and spleens were harvested after treatment.

16. Statistical Analysis

GraphPad Prism 9 (GraphPad, La Jolla, CA, USA) was used for statistical analysis. Data were presented as mean ± SD from at least 3 independent experiments of biological replicates, if not stated in the figure legend. Data were analyzed using two-sided Student's t-test when two groups were being compared. One-way or two-way analysis of variance (ANOVA) was used when more than two groups were compared (multiple comparisons). The difference was regarded as significant when the *p* value was less than or equal to 0.05. $*p < 0.05$, $**p < 0.01$, $***p < 0.001$, $****p < 0.0001$, ns, not significant.

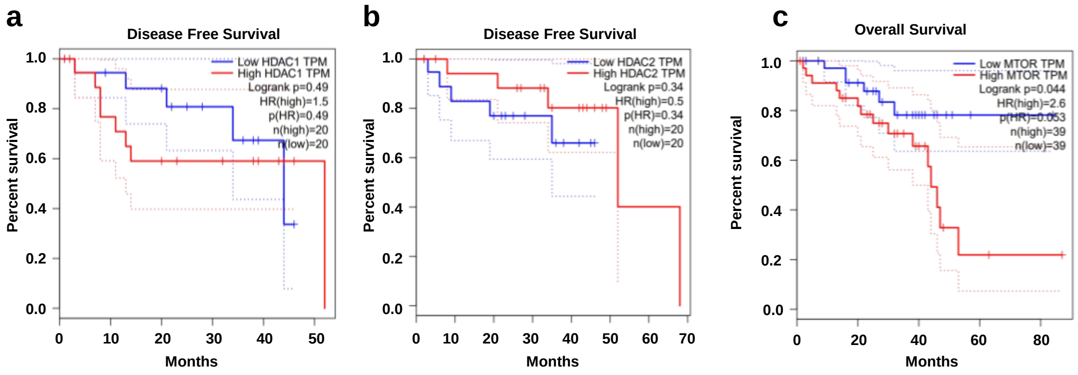

Figure S1. Correlation of disease-free survival with **a)** HDAC1, **b)** HDAC2 expression in uveal melanoma patients. **c)** The correlation of overall survival with mTOR expression in patients with UVM. Data in a-c) were analyzed using GEPIA (http://gepia.cancer-pku.cn/).

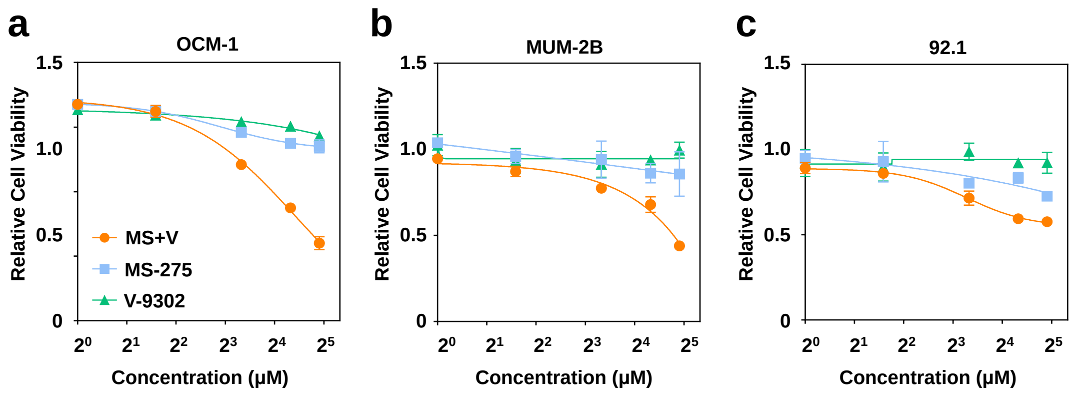

Figure S2. Relative cell viabilities of OCM-1, MUM-2B and 92.1 cells with 24 h treatment of MS-275, V-9302 and MS-275+V-9302 *via* MTT assay, respectively.

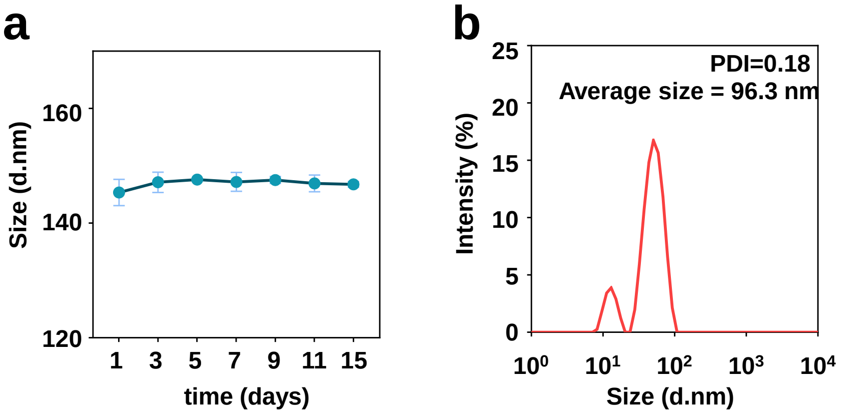

Figure S3. a) The size distribution throughout 15 days of storage in serum by DLS. **b)** Hydrodynamic diameters of NPs in 10 mM H_2O_2 measured by DLS.

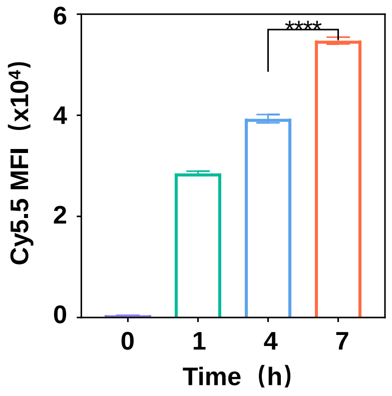

Figure S4. Quantification of intracellular uptake of Cy5.5 labeled NPs. Data were from three independent experiments. Datas were presented as mean ± SD. Statistical significances between every two groups were calculated *via* one-way ANOVA. ****$p < 0.0001$.

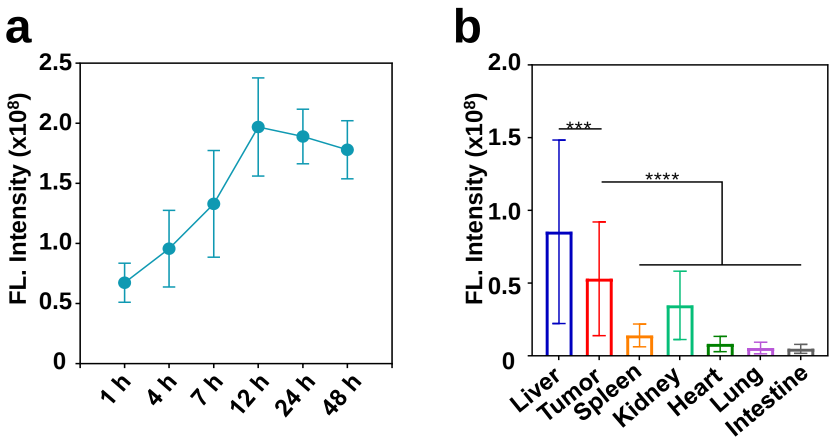

Figure S5. a) Quantification of fluorescence intensity in mice at different time points, **b)** Mean fluorescence intensity of NPs-Cy7.5 in major organs and tumor for 48 h after intravenous injection. Statistical significance between all groups was calculated *via* one-way ANOVA. ***$p < 0.001$, ****$p < 0.0001$.

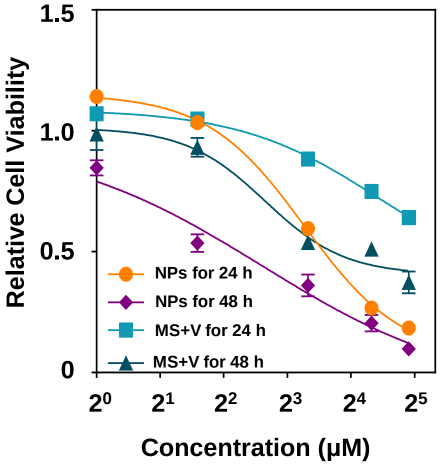

Figure S6. Relative cell viabilities of OCM-1 with treatment of NPs for 24 h, NPs for 48 h, MS+V for 24 h, MS+V for 48 h *via* MTT assay, respectively.

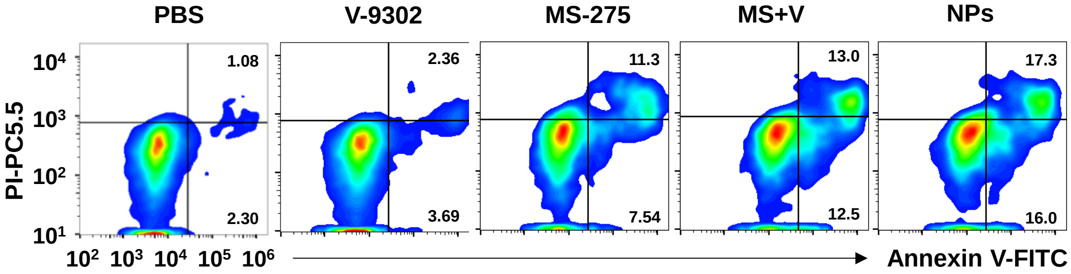

Figure S7. Flow cytometric profiles of apoptotic ratio via FCM. All groups were treated with the following compound concentration for 24 hours: PBS, MS-275 (10 μM), V-9302 (2 μM), MS-275 (10 μM)+V-9302 (2 μM) or NPs (10 μM).

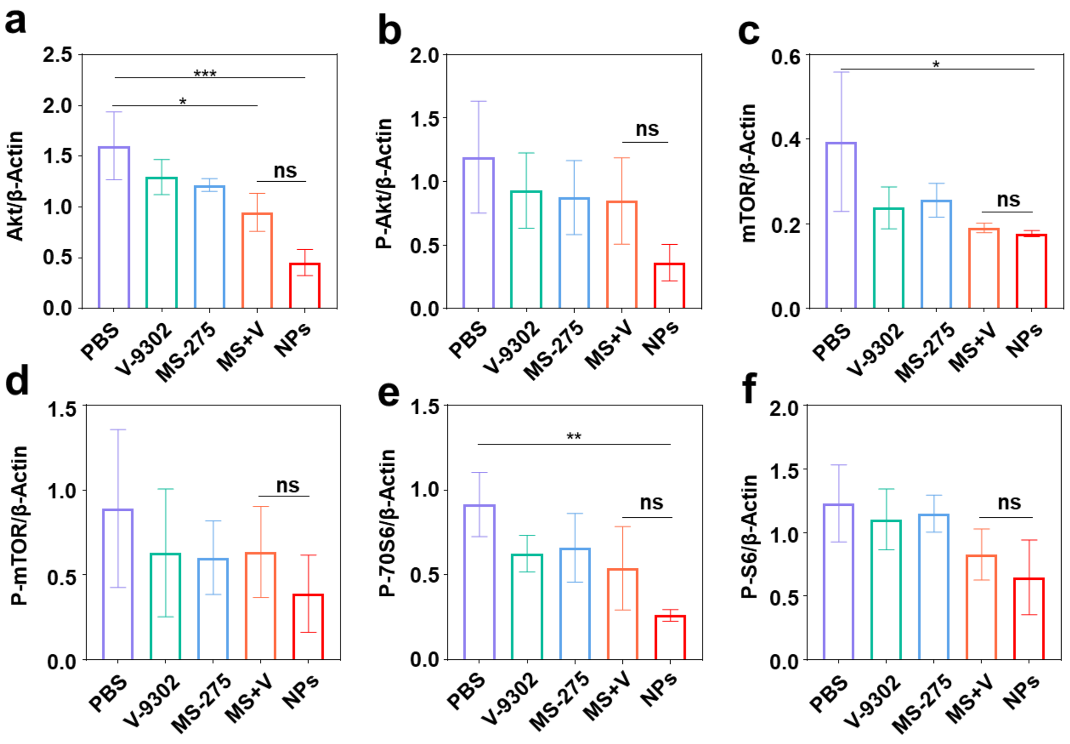

Figure S8. Relative expression of proteins in the mTOR pathway. **a)** Relative expression of Akt. **b)** Relative expression of P-Akt. **c)** Relative expression of mTOR. **d)** Relative expression of P-mTOR. **e)** Relative expression of P-70S6. **e)** Relative expression of P-S6. β-Actin was used as the internal reference protein. Data were from three independent experiments. Datas were presented as mean ± SD. Statistical significance between all groups was calculated *via* one-way ANOVA. **p* < 0.05, ***p* < 0. 01, ****p* < 0.001, ns, not significant.

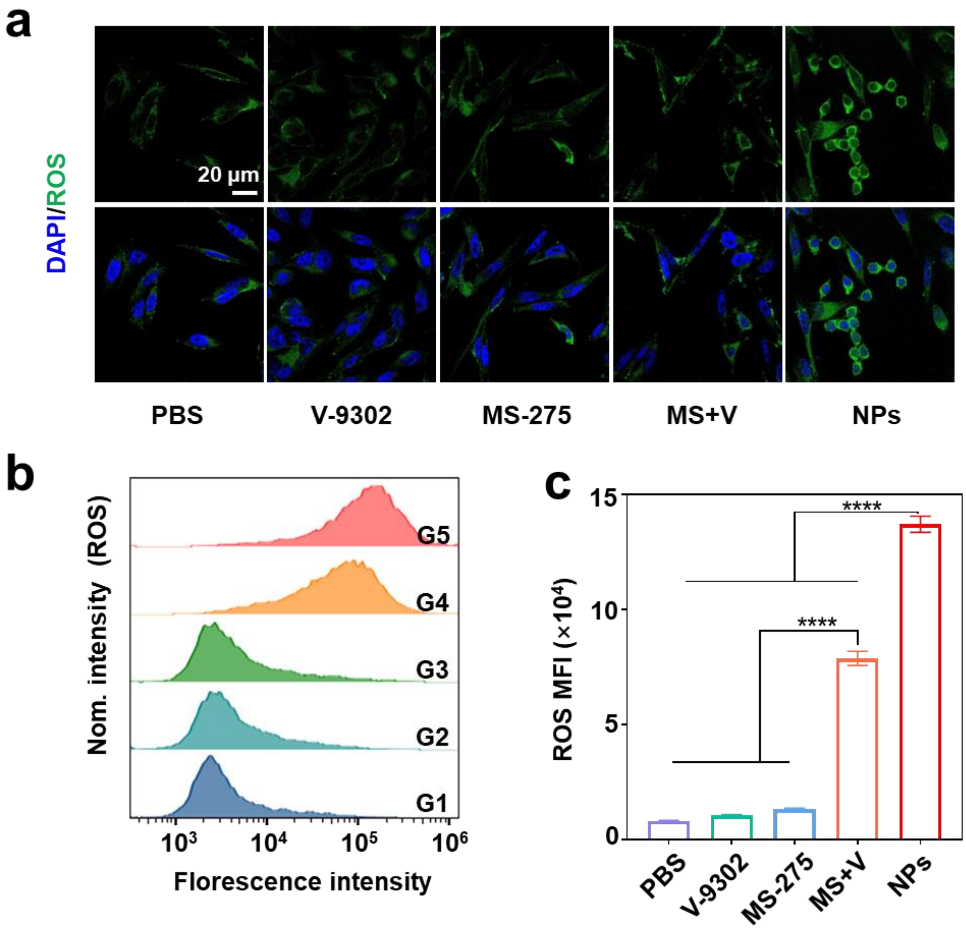

Figure S9. a) CLSM images of ROS in cells after various treatments. (blue, DAPI; green, ROS). **b)** Flow cytometric profiles and **c)** Quantification of ROS in OCM-1 cells for 24 h treatment of MS-275, V-9302, MS-275+V-9302 and NPs. Data were from three independent experiments. Datas were presented as mean ± SD. Statistical significance between all groups was calculated *via* one-way ANOVA. ****$p < 0.0001$.

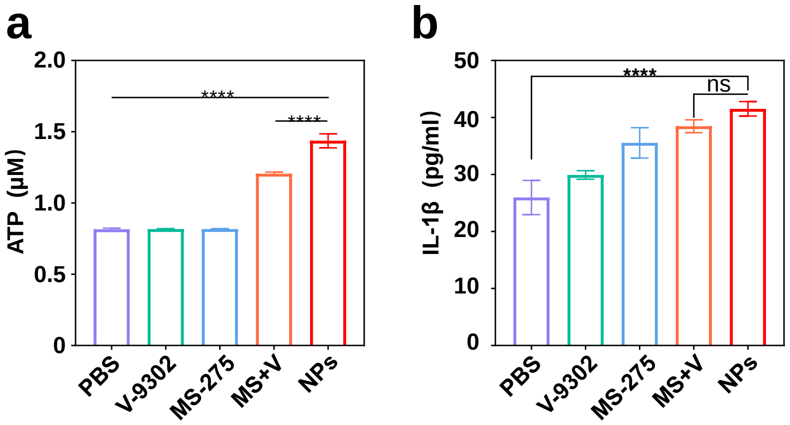

Figure S10. a) Quantification of ATP in OCM-1 cells supernatant after 24 h treatment of MS-275, V-9302, MS-275+V-9302 and NPs. **b)** Quantification of IL-1β in OCM-1 cells supernatant after 24 h treatment of MS-275, V-9302, MS-275+V-9302 and NPs. Data were from three independent experiments. Datas were presented as mean ± SD. Statistical significance between all groups was calculated *via* one-way ANOVA. ****$p < 0.0001$, ns, not significant.

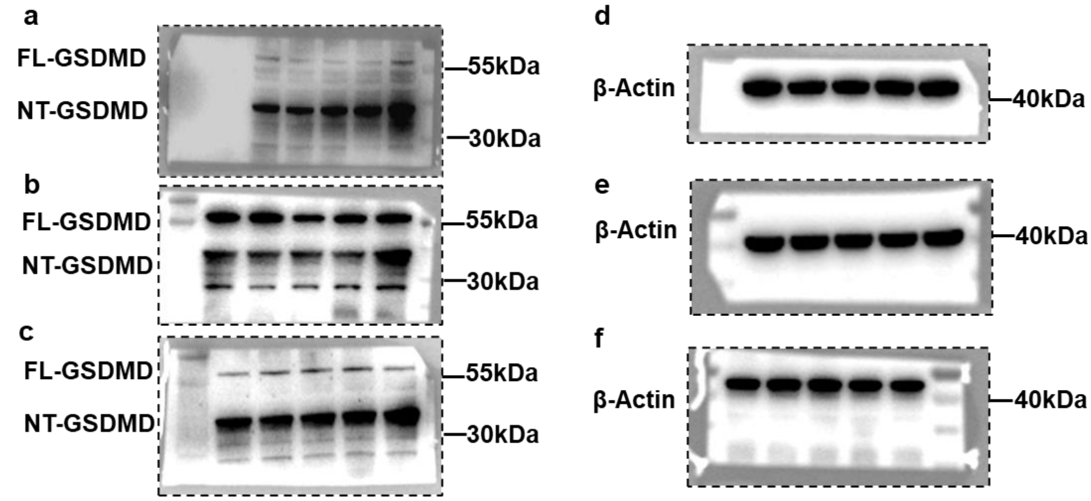

Figure S11. Original western blot images of **a, b, c)** full length of GSDMD (FL-GSDMD), N-terminal of GSDMD (NT-GSDMD) and **d, e, f)** β-Actin after different treatments for 24 h in OCM-1 cells. **Figures S11a** and **S11e** are used in **Figure 4h** in the main text.

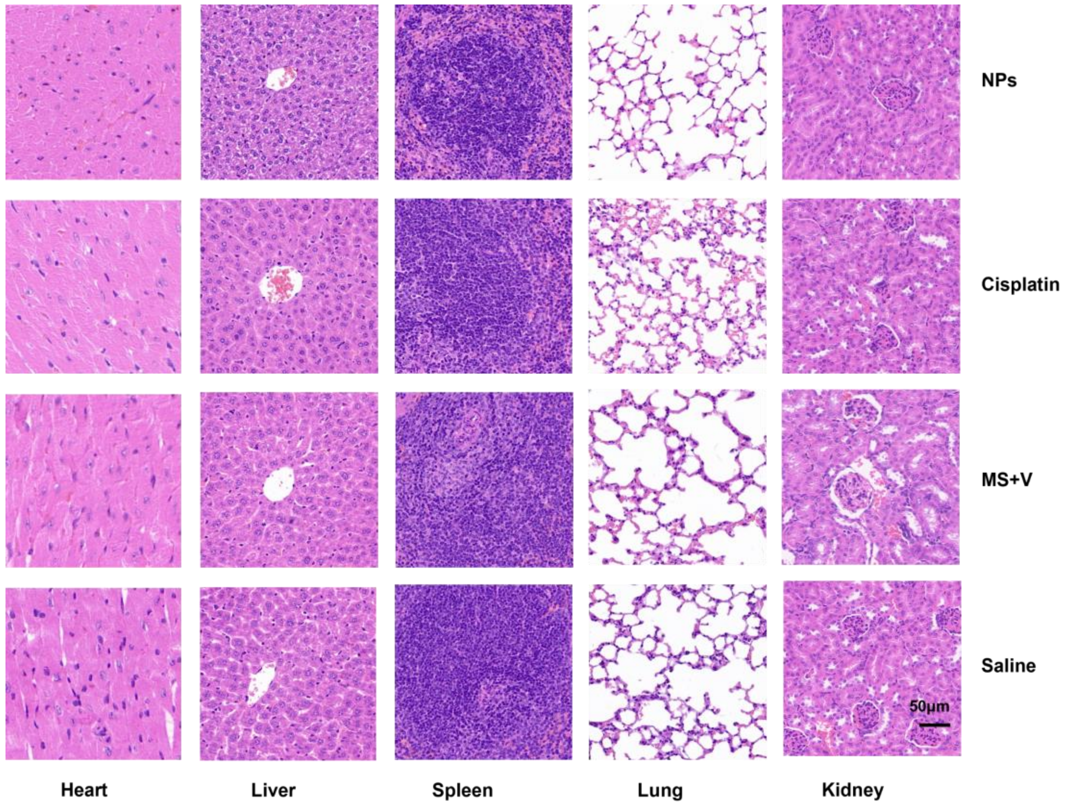

Figure S12. H&E staining of major organs (heart, liver, spleen, lung, and kidney) of BALB/c nude mice treated with saline, MS+V, cisplatin, or NPs at 4 mg/kg body weight. n = 5 mice per group. In the heart sections of each group, the cardiomyocytes were neatly arranged, the distribution of nuclei was centered and clear, and the morphology was complete. In liver tissue sections of each group, the structure of the liver lobules was clear, and the hepatocytes were arranged in a radial pattern centered on the central vein to form the liver plate. In the spleen sections of each group, the structure of the germinal center was complete, and the cells were neatly arranged. In the lung tissue sections of each group, the shape of alveoli was intact, no obvious thickening of alveolar wall was seen, and a few red blood cells were seen in the alveoli. In the kidney tissue sections of each group, the glomerular structure was intact, and no obvious degeneration or edema was seen in the renal interstitium.

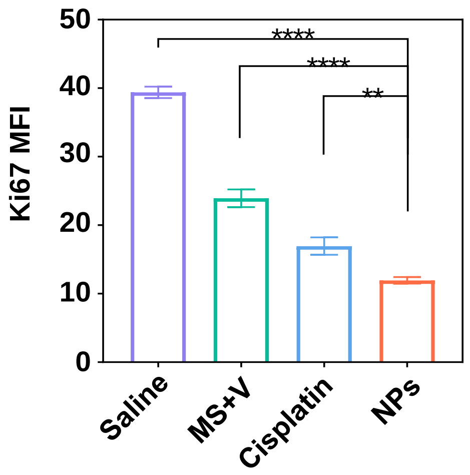

Figure S13. Relative MFI of Ki67 in tumor tissues. Data are presented as mean ±SD. Statistical significance between all groups was calculated *via* one-way ANOVA. $^{**}p < 0.01$, $^{****}p < 0.0001$.

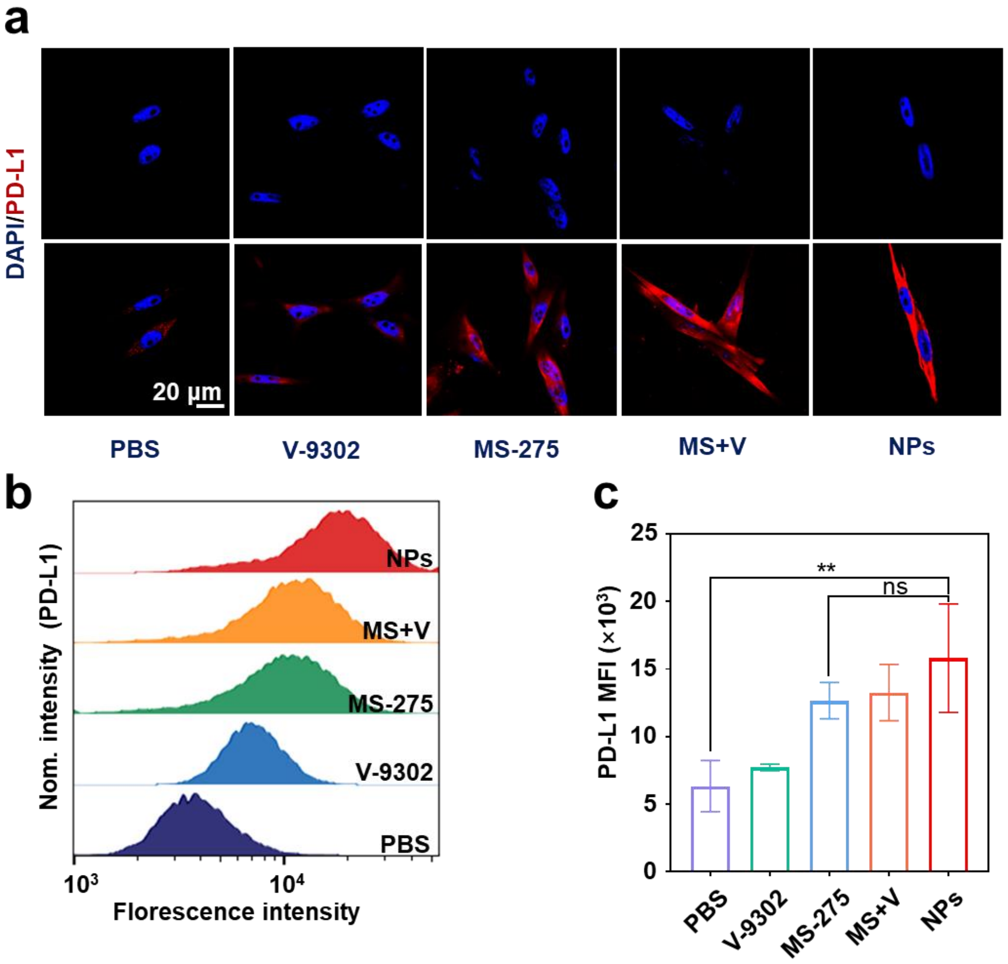

Figure S14. a) CLSM images of PD-L1 in OCM-1 cells treated with MS-275, V-9302, MS-275+V-9302 or NPs. **b)** Flow cytometric profiles and **c)** Quantification of PD-L1 in OCM-1 cells treated with MS-275, V-9302, MS+V or NPs. Data were from three independent experiments. Datas were presented as mean ± SD. Statistical significance between all groups was calculated *via* one-way ANOVA. $^{**}p < 0.01$, ns, not significant.

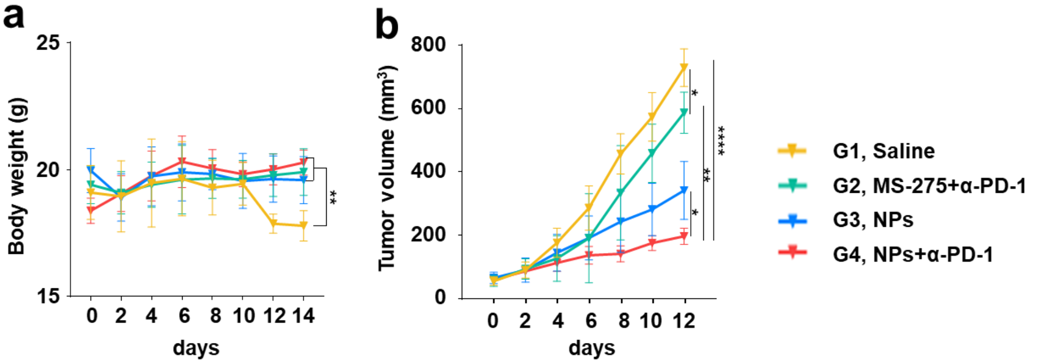

Figure S15. a) Body weight changes and **b)** Tumor growth inhibition curves of mice treated with Saline, MS-275 (4 mg /kg body weight, intravenously) +α-PD-1 (10 mg /kg body weight, intraperitoneal), NPs (4 mg /kg body weight, intravenously) and NPs (4 mg /kg body weight, intravenously) +α-PD-1(10 mg /kg body weight, intraperitoneal). n = 5 mice per group. Statistical significance between all groups was calculated *via* two-way ANOVA. *$p < 0.05$, **$p < 0.01$, ****$p < 0.0001$.

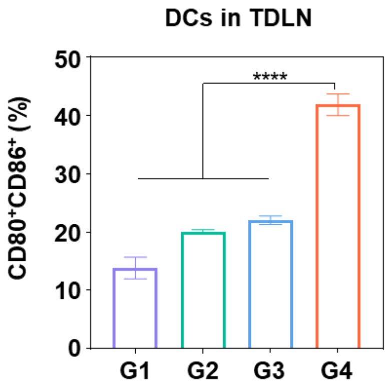

Figure S16. The percentage of mature DCs populations ($CD80^{+}CD86^{+}$) within the TDLNs in each group are presented as histograms. Statistical significance between all groups was calculated *via* one-way ANOVA. ****$p < 0.0001$.

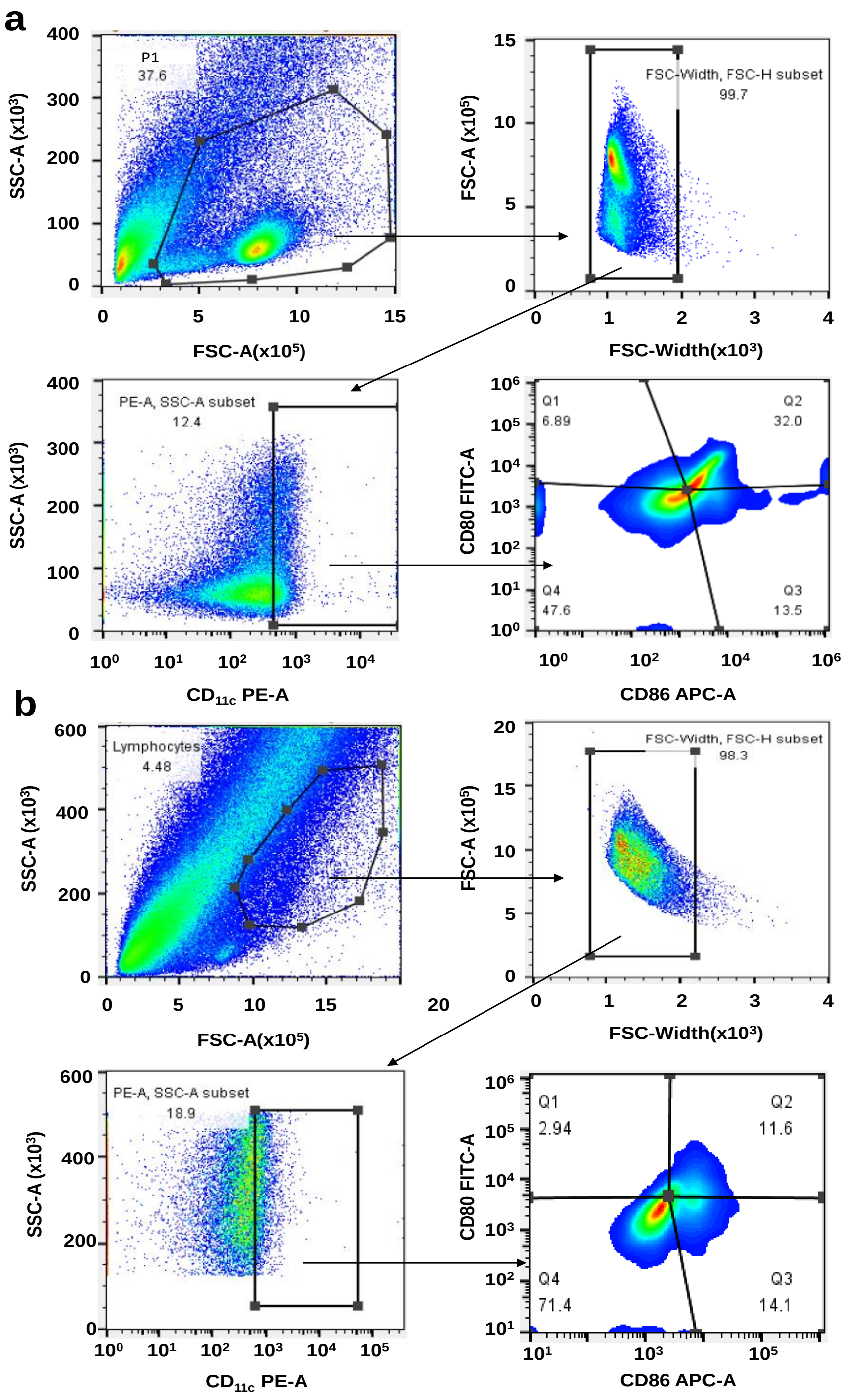

Figure S17. The gating strategy of $CD80^{+}CD86^{+}$ cells in **a)** TDLNs and **b)** tumors for flow cytometric analysis.

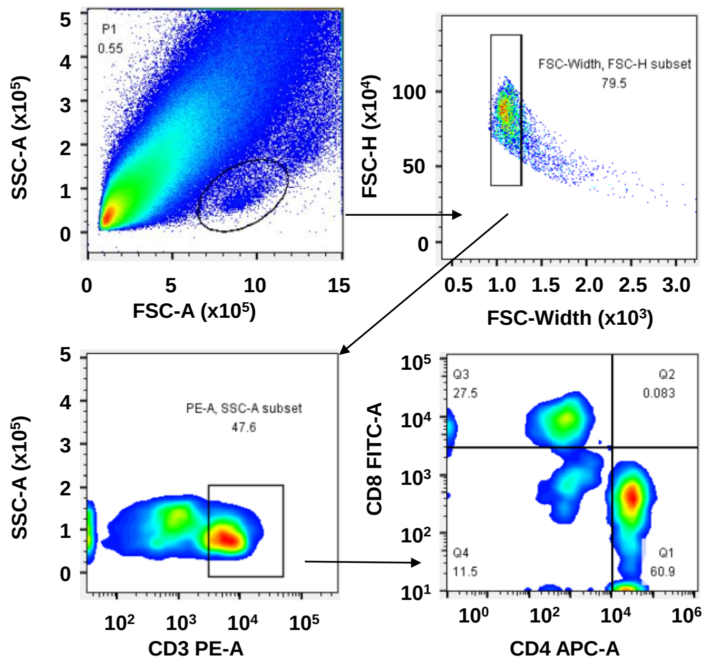

Figure S18. The gating strategy of $CD3^+CD8^+$ cells in tumor for flow cytometric analysis.

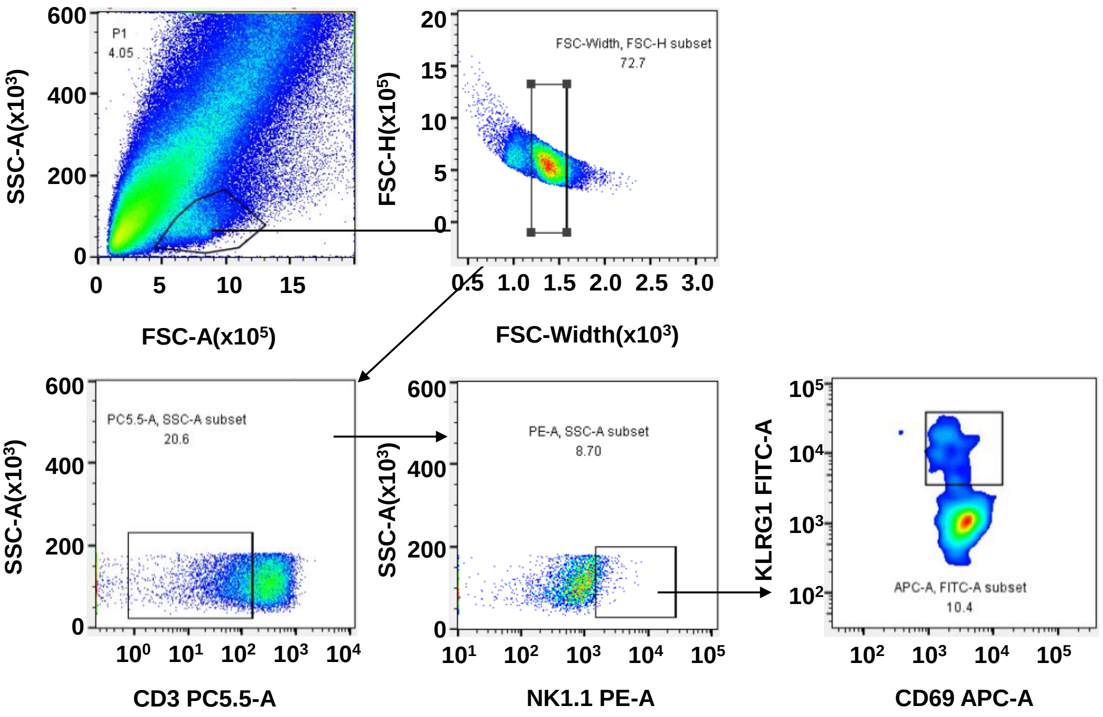

Figure S19. The gating strategy of NK ($CD3^{-}NK1.1^{+}CD69^{+}KLRG1^{+}$) cells in tumor for flow cytometric analysis.

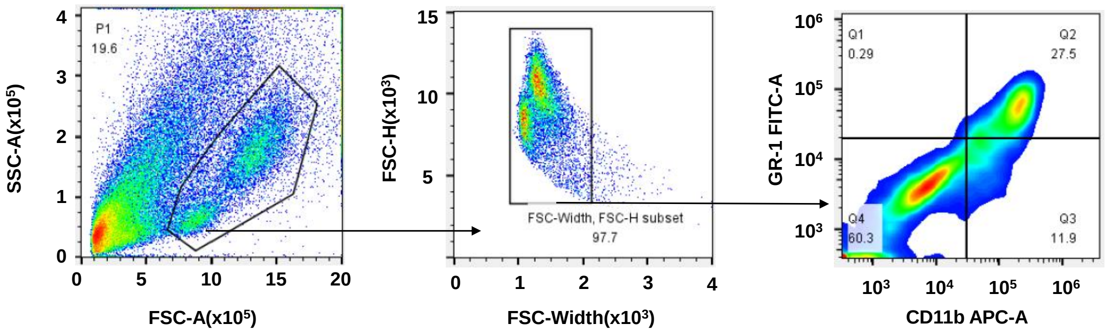

Figure S20. The gating strategy of MDSC ($CD11b^+GR\text{-}1^+$) cells in tumor for flow cytometric analysis.

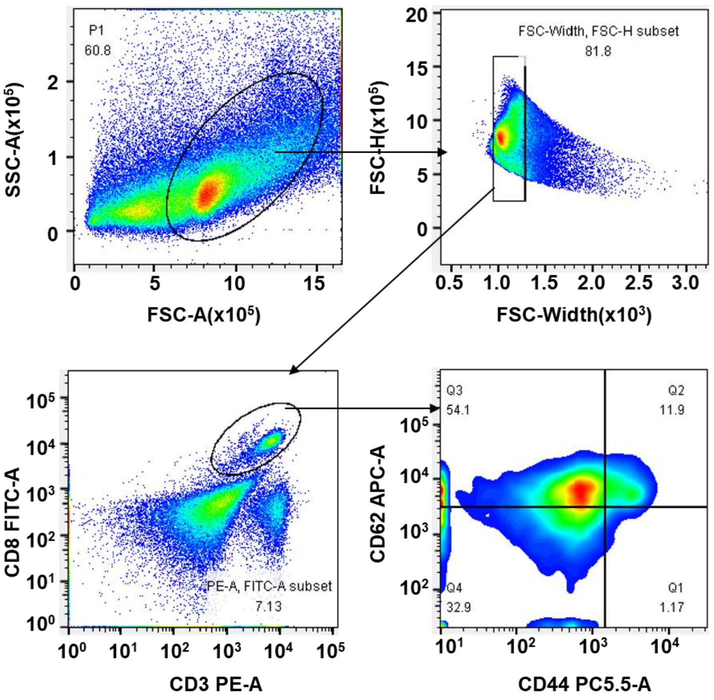

Figure S21. The gating strategy of T_{CM} ($CD44^{+}CD62L^{+}$) / T_{EM} ($CD44^{+}CD62L^{-}$) cells in spleen for flow cytometric analysis.

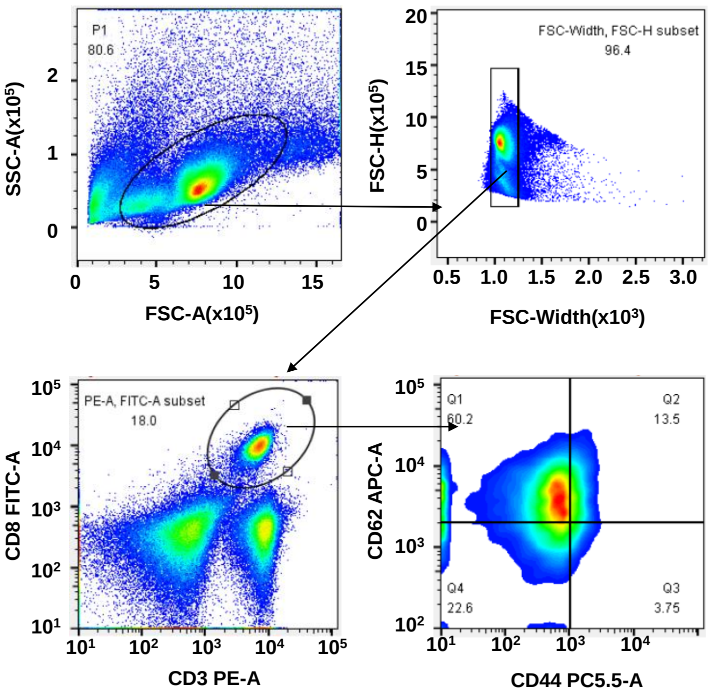

Figure S22. The gating strategy of T_{CM} ($CD44^{+}CD62L^{+}$) cells in TDLNs for flow cytometric analysis.

Table S1. IC_{50} (95%CI) of NPs for OCM-1, MUM-2B and 92.1 cell lines.

Cell line	IC_{50} of NPs（μM, 95%CI）
**OCM-1**	**9.63（9.01-10.42）**
**MUM-2B**	**25.57（23.71-27.48）**
**92.1**	**17.80（12.29-20.46）**

* IC_{50}, half maximal inhibitory concentration.

Table S2. IC_{50} (95%CI) of OCM-1 with treatment of NPs for 24 h, NPs for 48 h, MS+V for 24 h and MS+V for 48 h.

Treatment	IC50 (μM, 95%CI)
**NPs for 24 h**	**9.63 (9.01-10.42)**
**NPs for 48 h**	**6.06 (5.93-6.98)**
**MS+V for 24 h**	**21.40 (13.40-29.40)**
**MS+V for 48 h**	**6.29 (4.64-9.72)**

* IC_{50}, half maximal inhibitory concentration.

Table S3. Independent differential genes between NPs and MS+V.

Gene ID	Gene Symbol	Type	$\log_2$ (NPs / MS+V)	Q value (NPs / MS+V)
1299	'COL9A3'	mRNA	0.233341	1.69E-04
25829	'TMEM184B'	mRNA	0.181242	7.89E-04
131601	'TPRA1'	mRNA	0.331089	0.001268
642778	'NPIPA3'	mRNA	-1.61613	0.001977
6450	'SH3BGR'	mRNA	-1.68734	0.003896
1831	'TSC22D3'	mRNA	0.285657	0.003993
79090	'TRAPPC6A'	mRNA	0.774463	0.004638
1.01E+08	'POC1B-GALNT4'	mRNA	-1.0961	0.011769
3706	'ITPKA'	mRNA	-0.77248	0.013495
262	'AMD1'	mRNA	-0.14316	0.013786
80317	'ZKSCAN3'	mRNA	-0.42947	0.017472
22993	'HMGXB3'	mRNA	0.141146	0.019538
28962	'OSTM1'	mRNA	0.139981	0.023394
55075	'UACA'	mRNA	-0.16424	0.023646
85315	'PAQR8'	mRNA	0.190119	0.025064
83937	'RASSF4'	mRNA	0.227526	0.026482
153129	'SLC38A9'	mRNA	0.374593	0.026498

Gene ID	Gene Symbol	Type	$\log_2$ (NPs / MS+V)	Q value (NPs / MS+V)
51067	'YARS2'	mRNA	-0.26626	0.028623
55037	'PTCD3'	mRNA	-0.16436	0.031611
5908	'RAP1B'	mRNA	-0.13514	0.034981
55283	'MCOLN3'	mRNA	0.331429	0.035347
9774	'BCLAF1'	mRNA	-0.1457	0.042656
54946	'SLC41A3'	mRNA	0.236892	0.042924
388341	'LRRC75A'	mRNA	0.258742	0.044088
3133	'HLA-E'	mRNA	0.112093	0.045149
79041	'TMEM38A'	mRNA	0.342418	0.04846
57519	'STARD9'	mRNA	0.208598	0.049907

Reference:

[1] F. Ding, F. Li, D. Tang, B. Wang, J. Liu, X. Mao, J. Yin, H. Xiao, J. Wang, Z. Liu, Angew Chem Int Ed 2022, 61, DOI 10.1002/anie.202203546.
